# Consequences of tropical land use for multitrophic biodiversity and ecosystem functioning

**DOI:** 10.1038/ncomms6351

**Published:** 2014-10-28

**Authors:** Andrew D. Barnes, Malte Jochum, Steffen Mumme, Noor Farikhah Haneda, Achmad Farajallah, Tri Heru Widarto, Ulrich Brose

**Affiliations:** 1 Department of Systemic Conservation Biology, J.F. Blumenbach Institute of Zoology and Anthropology, University of Göttingen, Berliner Strasse 28, 37073 Göttingen, Germany; 2 Department of Silviculture, Faculty of Forestry, Bogor Agricultural University, Darmaga Campus, Bogor 16680, Indonesia; 3 Department of Biology, Faculty of Mathematics and Natural Sciences, Bogor Agricultural University, Darmaga Campus, Bogor 16680, Indonesia

## Abstract

Our knowledge about land-use impacts on biodiversity and ecosystem functioning is mostly limited to single trophic levels, leaving us uncertain about whole-community biodiversity-ecosystem functioning relationships. We analyse consequences of the globally important land-use transformation from tropical forests to oil palm plantations. Species diversity, density and biomass of invertebrate communities suffer at least 45% decreases from rainforest to oil palm. Combining metabolic and food-web theory, we calculate annual energy fluxes to model impacts of land-use intensification on multitrophic ecosystem functioning. We demonstrate a 51% reduction in energy fluxes from forest to oil palm communities. Species loss clearly explains variation in energy fluxes; however, this relationship depends on land-use systems and functional feeding guilds, whereby predators are the most heavily affected. Biodiversity decline from forest to oil palm is thus accompanied by even stronger reductions in functionality, threatening to severely limit the functional resilience of communities to cope with future global changes.

## Abstract (web summary)

Transformation of natural ecosystems into agricultural land is usually accompanied by extensive biodiversity loss. Calculating multitrophic energy fluxes, Barnes *et al.* report severe reductions of biodiversity and ecosystem functioning from tropical rainforest to oil-palm plantations.

## Graphical abstract

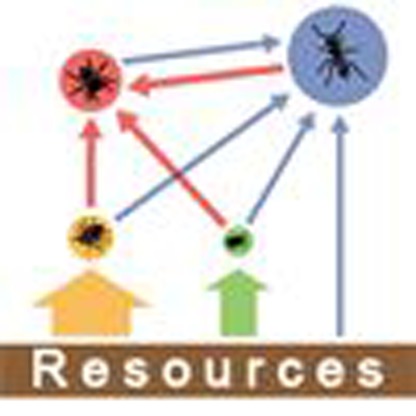

The transformation from natural ecosystems to agricultural land use and its continued intensification has led to extensive losses in biodiversity and ecosystem services^1^ resulting in the degradation of human well being^2^. The transformation of lowland tropical rainforest to oil palm (*Elaeis guineensis* Jacq.) plantations has gained more recent attention as an especially severe threat to tropical biodiversity^3^,^4^. In the last 25 years the total plantation area of oil palm has tripled, with current global estimates of over 15 million hectares^3^, making this crop one of the world’s most rapidly expanding forms of agriculture^5^. It is now clear that the expansion of oil palm agriculture is one of the greatest causes of deforestation^6^,^7^, and this threat appears to be increasing without respite as Indonesia, one of the world’s leaders in oil palm, makes plans to double production by 2020 (ref. 8). The rapid expansion of such large-scale land-use transformation raises questions about the impending implications for biodiversity and ecosystem functioning in the tropics.

Despite a broad consensus that biodiversity is positively correlated with ecosystem functioning in controlled experiments^9^,^10^, there are few real-world examples of such biodiversity–ecosystem functioning relationships^11^,^12^. In fact, until now there have been no studies that explore the relationship between biodiversity and ecosystem functioning in ecosystems undergoing agricultural land-use transformation to oil palm. Thus, our knowledge of this globally important land-use conversion is strongly limited. Furthermore, over the past decade there have been important advances towards multitrophic approaches in research investigating biodiversity–ecosystem functioning relationships^9^,^13^,^14^,^15^,^16^. Despite these advances, however, we are still substantially limited by the lack of clear approaches to quantify single measures of ecosystem functioning that can be compared among any combination of trophic levels. This has resulted in our inability to directly look at whole-community relationships between entire species assemblages and the respective functional processes carried out in these communities.

Here we use the total energy flux between functional feeding guilds as a measure of multitrophic ecosystem functioning, as many studies have suggested process rates, such as energy fluxes, to be important proxies for ecosystem functioning^10^,^13^,^17^. Depending on the resource pool that the energy flux comes from, these fluxes can be directly related to ecosystem services such as decomposition^18^,^19^, plant biomass production^20^,^21^ or biocontrol through predation^22^. These energy flux calculations are based on metabolic scaling theory^23^ and principles of food-web energy dynamics^18^. Using individual metabolic rates that are dependent on body mass, environmental temperature and phylogenetic grouping^18^,^24^, combined with resource-specific assimilation efficiencies^25^ and energy loss to predation^18^, we present this energy flux calculation as a unified measure of multitrophic ecosystem functioning (Fig. 1). Studies that incorporate diversity across trophic levels to test the relationship between biodiversity and ecosystem functioning have predominantly used only biomass as the measure of ecosystem function^26^. However, the metabolic activity and thus the energy-processing rates of these biomass pools can vary substantially. Integrating over body mass, phylogeny and temperature with their constraints on metabolic rates, and additionally taking into account assimilation efficiencies and loss to predation, our measure of whole-community energy flux inherently incorporates not only biomass but also other important ecosystem attributes enabling the quantification of emergent functional properties of ecosystems that would otherwise remain undetected. As such, our measure of energy flux provides a comprehensive and robust measure of multitrophic ecosystem functioning that can be utilized for modelling biodiversity–ecosystem functioning relationships for any assemblage of taxonomic groups, while incorporating multiple ecological functions.

In the tropical lowland rainforests of Sumatra, Indonesia, which have been undergoing vast land-use transformation to oil palm^7^, we quantify the impacts of this transformation ranging from tropical secondary rainforest, jungle rubber and intensively managed rubber, to oil palm. We utilize data gathered from 32 sites in Sumatra, Indonesia, comprising 2,415 populations of 871 species. First, we investigate the biodiversity value of jungle rubber, conventional rubber and secondary forest compared with oil palm agriculture by comparing observed species richness, density and biomass of litter-associated macroinvertebrate communities across these systems. Second, as a multitrophic measure of the rate of ecosystem processes carried out by these communities, we calculate total solid fresh mass energy flux in a system by incorporating community metabolism^27^, resource-specific assimilation efficiencies and biomass loss to predation^18^ into whole-community energy flux equations (Fig. 1). This provides a quantitative measure of multitrophic ecosystem functioning, defined here as the total flux of energy from any resource pool to consumer trophic levels. In addition, this measure can be attributed to specific functional feeding guilds within communities to look for patterns in ecosystem functioning at different trophic levels. Using the energy-mass flow conversion^28^, we express energy flux as kilograms per hectare, per year and explore the relationship between total species diversity and energy flux, distinguishing among four transformation systems to test for land-use-dependent biodiversity–ecosystem functioning relationships. Our results demonstrate strong losses in species diversity that, in turn, predict reductions in whole-community energy fluxes. However, these reductions are strongest in oil palm systems, suggesting that land-use conversion from forest to oil palm causes disproportionally strong losses in multitrophic ecosystem functioning.

## Results

### Transformation to oil palm leads to biodiversity loss

Using generalized linear mixed effects models, we show that transformation of tropical rainforest to oil palm plantations leads to severe losses in species richness (45% decline), animal density (48% decline) and biomass (52% decline; Fig. 2a–c and Supplementary Table 1), supporting previous studies suggesting that land-use transformation to oil palm poses one of the greatest threats to global biodiversity^3^. Beyond mere diversity effects, land-use transformation altered animal densities and biomass, threatening to not only drive species extinctions but also to eliminate vital ecological functions. The effects of land-use transformation on species richness and animal densities were additionally dependent on functional feeding guilds, with predators decreasing in species richness and density most rapidly (Fig. 2a–c and Supplementary Table 1) as could be expected for higher trophic level feeding guilds^29^. Such alteration of higher trophic levels is likely to have severe indirect functional impacts on other functional guilds within the trophic network^30^.

### Community metabolism

Summing up individual metabolic rates, we demonstrate that transformation of forest to oil palm yields a 51% decrease in community metabolism, with jungle rubber and rubber only 16% and 10% below forest levels of community metabolism, respectively. However, all systems yielded significantly higher community metabolism than oil palm (Fig. 2d and Supplementary Table 1). As such, we show that ecosystem energy processing is critically reduced in oil palm plantations. Interestingly, biomass responses to land-use transformation among feeding guilds were not clearly comparable to responses in community metabolism (Fig. 2c,d). This suggests that systematic changes in species composition, body-mass distributions (Supplementary Fig. 1) and biomass exhibited a complex interaction in determining the functional consequences of land-use transformation.

### Whole-community energy fluxes and ecosystem functioning

**Figure 1:**
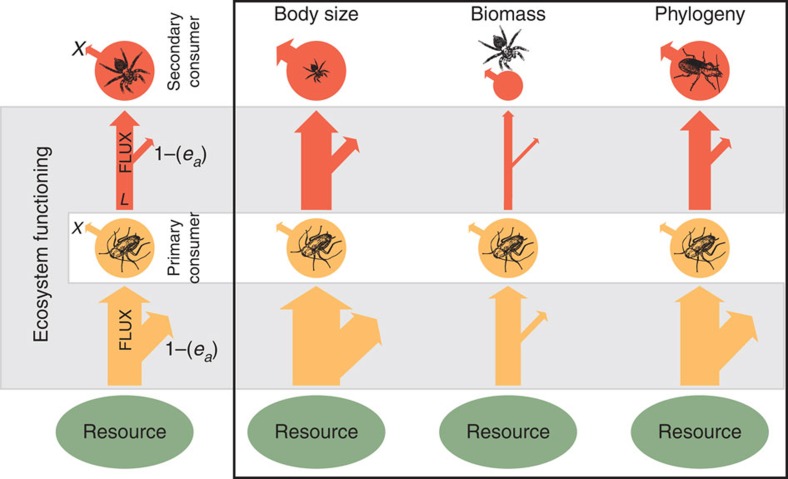
Energy fluxes along a conceptual food chain as a measure of multitrophic ecosystem functioning. Energy flux between two nodes is calculated as 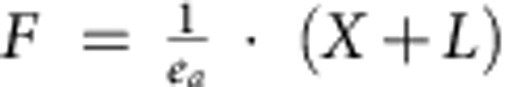, where *F* is the total energy flux into the network node of a feeding guild (vertical red and yellow arrows), *e*_a_ is the diet-specific assimilation efficiency (denoted by diagonal arrows arising from the flux arrows), *X* is the per-unit-mass metabolic demand of the feeding guild (which is nonlinearly dependent on body sizes, temperature and phylogeny) and *L* is the loss to predation from the node (for the yellow node, this is equal to the flux to the red secondary consumer node). Here we demonstrate three examples where changes in the mean body size (size of black animal icons), biomass (diameter of red and yellow circles) or phylogeny (black animal icons) on any trophic level (here demonstrated by the secondary consumer guild) can result in nonproportionally altered total energy flux (sum of all arrow widths in the food chain).

**Figure 2:**
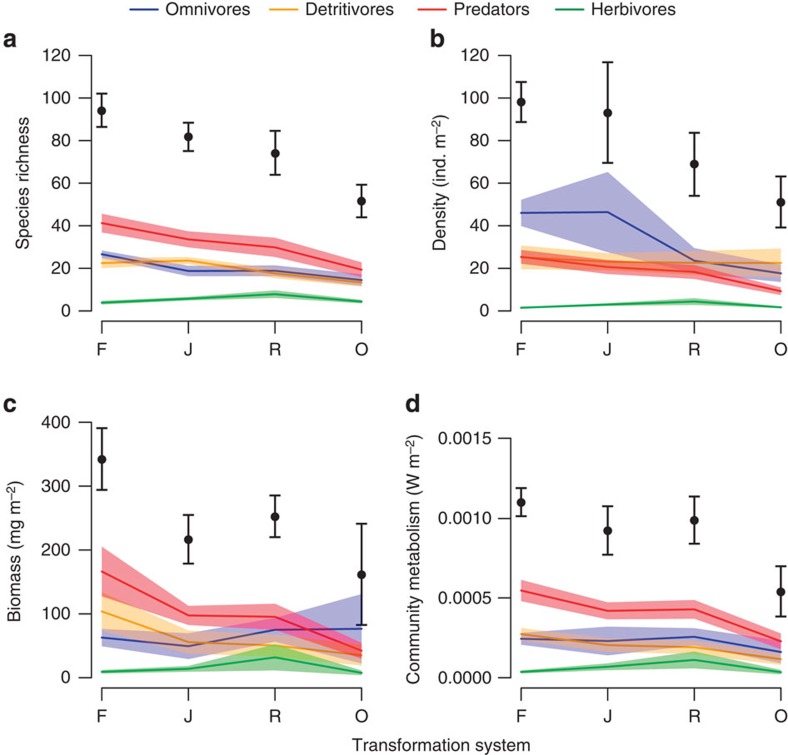
Effects of land-use transformation on macroinvertebrate communities. The mean (±s.e., *n*=32) species richness (**a**), density (**b**), biomass (**c**) and community metabolism (**d**) of the total community (black points) and of each functional feeding guild (coloured lines) for the four land-use transformation systems: forest (F), jungle rubber (J), rubber (R) and oil palm (O).

**Figure 3:**
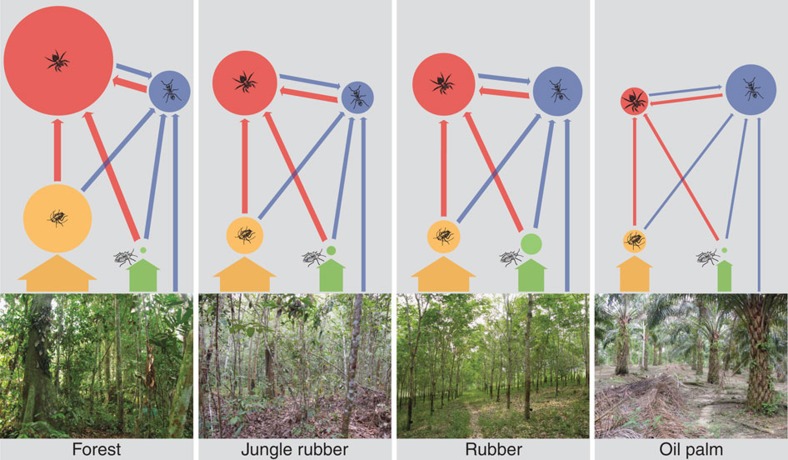
Effects of land-use transformation on community energy networks. Energy networks displaying the relative annual energy flux (coloured arrow width weighted by calculated energy flux (kg ha^−1^ per year)) and biomass (coloured node diameter weighted by total biomass) among the functional feeding guilds: predators (red), omnivores (blue), detritivores (yellow) and herbivores (green). Each panel represents an energy network for one of the four land-use transformation systems.

Aiming to visualize the complex interplay between community biomass dynamics and energy flux, we constructed energy networks for the four transformation systems (Fig. 3) based on total energy fluxes as a promising way to quantify multitrophic ecosystem functioning (Fig. 1). In addition to the general decreases in biomass (node sizes in Fig. 3) and energy-processing rates (arrow widths in Fig. 3), we also found a systematic shift from predator to omnivore dominance when comparing forest and oil palm systems. Specifically, we found that predator biomass in oil palm yielded only 25% of their biomass in forest (0.424 and 1.664 kg ha^−1^, respectively), while the predator-driven energy flux was reduced to 46% of the energy flux driven by predators in forest (30.697 and 66.816 kg ha^−1^ per year, respectively). In contrast, omnivore biomass in oil palm was 22% higher than in the forest (0.767 compared with 0.629 kg ha^−1^), while omnivore-driven energy flux in the oil palm was 47% lower than in forest communities (32.531 compared with 61.900 kg ha^−1^ per year; Supplementary Table 2), suggesting a considerable mismatch of biomass and energy flux, partly dependent on the trophic group in question. In our analyses, this disparity finds its explanation in varying body-mass distributions (Supplementary Fig. 1) and assimilation efficiencies that strongly modify how biomass translates into total resource assimilation rates (Fig. 1). These results suggest that biomass, alone, may be an unsuitable proxy for general ecosystem functioning in animal communities.

### Multitrophic biodiversity-ecosystem function relationships

**Figure 4:**
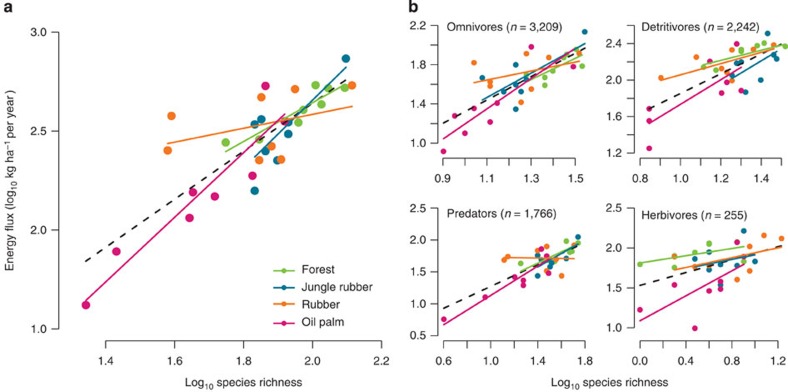
Relationship between species richness and community energy fluxes. Linear mixed effects models for (**a**) entire communities and (**b**) separated into functional feeding guilds. Black dashed lines denote overall model fits and coloured lines indicate different land-use transformation systems.

Until now, most studies investigating biodiversity–ecosystem function relationships have focused on single trophic levels^31^,^32^. We present a new approach to easily quantify multitrophic ecosystem functioning, requiring only information on body mass, phylogeny, temperature and assimilation efficiencies to overcome previous limitations in biodiversity–ecosystem functioning research. Utilizing this approach, we also investigated the relationship between species richness and ecosystem functioning, identifying a clear linear positive effect of diversity on total energy flux (Fig. 4a and Supplementary Table 3). The relationship between diversity and energy flux was dependent on land-use transformation system, whereby oil palm and jungle rubber showed the strongest decrease in energy flux per unit loss in species richness (Fig. 4a and Supplementary Table 3). Our results suggest that each loss of species in oil palm and jungle rubber therefore would be followed by proportionately higher losses in energy flux, compared with equal species losses in forest and rubber. We found the same pattern as in the overall trend for the predator group, which showed transformation system-dependent relationships between species richness and energy flux (Fig. 4b). However, for omnivores, detritivores and herbivores there was a linear effect of diversity on energy flux driven by these groups; however, this effect was independent of transformation system (Fig. 4b and Supplementary Table 3). This implies that studies focusing on single trophic levels, or even specific species, may fail to detect the alteration of ecosystem processes resulting from land-use transformation. These results call for a wider application of multitrophic approaches that not only measure one ecosystem property, such as total productivity or decomposition, but that also aim to assess whole-community ecosystem processes such as total energy flux.

## Discussion

Our study reflects previous findings that the transformation of forest systems to oil palm has severe impacts not only on single animal populations but also on communities as a whole. In particular, species richness and animal biomass are most significantly affected. Furthermore, jungle rubber and rubber appear to represent intermediate steps in land-use intensification. Their higher levels of biodiversity and ecosystem functioning indicate that they potentially provide higher ecological value than oil palm. As such, these rubber land-use systems could present economically viable, lower intensity land-use alternatives.

By taking a multitrophic ecosystem functioning approach we demonstrate that, at the community level, species loss leads to a direct linear decrease in ecosystem functioning. This means that any species loss will be followed by a proportionate loss in function, and this relationship becomes proportionately stronger in more intensive transformation systems such as oil palm plantations. Thus, every one of the few species in high-intensity land-use systems is functionally more important than species in low-intensity systems where functional redundancy is likely to be higher^33^. Without explicit consideration of multiple trophic levels, such emergent properties are likely to be overlooked. Our study demonstrates the crucial implications of tropical land-use intensification for biodiversity and ecosystem functioning across multiple trophic levels, suggesting that these globally important impacts will likely resonate beyond previously explored trophic boundaries.

## Methods

### Study site and sampling design

Sampling took place in the Jambi province of Sumatra, Indonesia, a region known as a hotspot for biodiversity, but that has also already undergone extensive deforestation^6^,^34^. In the second half of the last century, Sumatra’s forests have experienced vast transformation to rubber and oil palm monocultures^35^,^36^. This large-scale land-use conversion has left Sumatra with a very limited area of natural forest mainly restricted to national parks and even here, where logging has been reduced, it has not come to a complete halt^37^. This severe and extensive land-use transformation, that has progressed already further than in most other tropical landscapes, makes Sumatra a unique and ideal example system for studying the impacts of land-use conversion on biodiversity and ecosystem functioning.

We sampled secondary rainforest, jungle rubber, rubber and oil palm systems, replicated eight times across two landscapes (*n*=32; Supplementary Fig. 2). Sites were selected by first looking for landscapes in the Jambi province that still contained secondary rainforest. Second, we identified all lowland areas with little or no slope and then randomly selected two landscapes with 16 sites each. Among all of the 32 sampling sites, we maintained a minimum distance of 120 m to insure independence of the epigaeic invertebrate communities sampled. The secondary-forest regions lie within two protected areas, Bukit Duabelas National Park and Harapan Rainforest, and represent the least influenced land-use system. Jungle rubber—forest stands with a high percentage of rubber trees that are still regularly harvested—represents a low-impact agroforestry system^38^. Rubber and oil palm plantations serve as locally common^36^ high-impact monocultures. The 32 sites were carefully selected so that they were all of a similar age and from equal elevations close to the sea level. All agricultural systems (jungle rubber, rubber and oil palm) were treated and harvested by their owners with intensities typical for the respective transformation system.

### Animal sampling and calculation of response variables

Animal sampling took place between early October and early November 2012. All organisms were collected based on Permit No. 51/KKH-5/TRP/2014 issued by the Indonesian Institute of Sciences and the Ministry of Forestry. In all 32 of the 50 × 50-m sites, we sampled once in each of three 5 × 5-m subplots by sieving the leaf litter from 1 m^2^ through a coarse sieve of 2-cm-width mesh. In all, 7,472 macroinvertebrates were hand-collected from the sieving samples and stored in 65% ethanol. Specimens were identified to morphospecies and assigned to one of four feeding guilds: omnivores, detritivores, predators and herbivores, based on morphology and literature.

As biodiversity studies always suffer from undersampling and correlation of sample size with species richness, we compared observed species richness to both extrapolated and rarefied species richness, calculated in the ‘vegan’ package in R^39^, to assess the accuracy of our species-sampling effort. To extrapolate sampled species richness, we used the nonparametric second-order jacknife estimator^40^ to calculate extrapolated species richness from the three 1-m^2^ subsamples at each of the 32 sites, revealing an estimated mean sampling coverage of 56% (s.d. of±2.393%) making the second-order jacknife estimator the most accurate extrapolation method^40^. In addition, we calculated sample-based rarefaction, whereby rarefaction curves were calculated for each of the 32 sampled sites and then cut off at the sample size of the smallest sample (40 individuals). Because of the very high attrition of data during the rarefaction procedure (a total of 6,192 out of 7,472 individuals, or 83%, were removed), the rarefied species richness yielded very little resemblance to observed species richness when comparing across transformation systems, resulting in almost no pattern of rarefied richness among transformation systems (Supplementary Fig. 3). The jacknife2-extrapolated species richness, however, was extremely closely correlated with observed species richness (Pearson’s *ρ*=0.993) patterns among transformation systems (Supplementary Fig. 3), suggesting that our observed species richness did in fact accurately capture realistic patterns in total species diversity across the land-use transformation systems.

For each of the 7,472 animals collected, we measured individual body length to an accuracy of 0.1 mm using stage micrometres. We then converted all measured individual body lengths to fresh body mass using length-mass regressions and, where necessary, dry mass-fresh mass relationships from the literature (Supplementary Table 4), yielding an estimated fresh mass in milligrams for every collected individual. Where family-specific relationships were not available or animal body lengths in our collection fell outside of the size ranges of published regressions, we then used regressions from higher-order taxonomic groupings. For heavily damaged individuals that could not be measured for body length, we assigned these individuals a fresh body mass from the median body mass of all animals from the same species or order where only one individual of that species was collected. We then calculated community biomass (mg fresh mass m^−2^) for each of the 32 communities by summing together all individual body masses calculated from length-mass regressions as derived from the individually measured body lengths.

We calculated individual metabolic rates for all 7,472 animals using body masses, temperature and phylogeny^24^ (Supplementary Table 5). Temperature was measured over a period of at least 2.5 months at 30-cm depth below the soil surface in each site and averaged for each transformation system in each of the two landscapes. From this, community metabolism was calculated by summing together all individual metabolic rates within each of the 32 sites, providing the total metabolic demand for each of the 32 communities. Using diet-specific assimilation efficiencies^25^, energy loss to predation and community metabolism, we analytically calculated energy fluxes for each of these communities^18^ using the formula


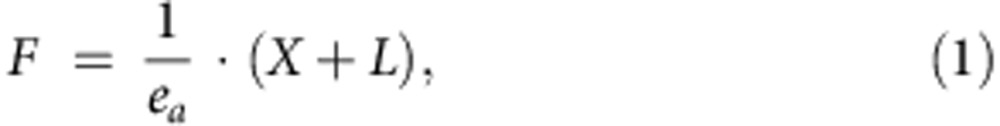


where *F* is the total energy flux into the network node of a feeding guild, *e*_*a*_ is the diet-specific assimilation efficiency, *X* is the metabolic demand of the feeding guild and *L* is the loss to predation that the feeding guild is subjected to (Fig. 1 and Supplementary Methods). In order to calculate the fluxes between the functional feeding guilds, we constructed a general network of feeding relationships (link structure in Fig. 3) that represents a null model for an energy network structure where no active preferences are assumed. We assumed that, of our four functional feeding guilds, energy fluxes to predators were split up equally into the three animal guilds below them. Energy fluxes to detritivores and herbivores were assumed to come from only detritus and plant material, respectively. Omnivores were assumed to receive energy in equal 25% proportions from the other three functional feeding groups (predators, detritivores and herbivores, making 75%) and the remaining 25% from both plant and detritus material combined (Supplementary Methods).

To assess how these assumptions of feeding preferences might affect the calculations of total energy fluxes, we reconstructed the energy networks so that omnivores were assumed to only consume plant and detritus material (50% derived from each) but with no energy derived from animal material. We then recalculated total energy fluxes and found an overall decrease of up to 54%, which appeared to be highly consistent among the different land-use transformation systems. This consistency between models was especially evident after calculating the loss of energy flux in the three agriculturally used systems compared with the forest system, demonstrating a maximum of only 3% disparity between the two models (Supplementary Fig. 4). This sensitivity analysis indicated that our presented method is highly robust in calculating differences in energy fluxes among different systems. Accordingly, the null model was accepted as the simplest model with the least diet preferences assumed. However, we still suggest that studies adopting this method of energy flux calculation should assign feeding preferences with caution, or employ other techniques such as stable isotope analysis to estimate feeding preferences.

### Statistical analyses

Using mixed effects models (GLMM’s), we tested the effects of ‘transformation system’ and its interaction with functional feeding guild on community responses, with ‘landscape’ as a random effect. ‘Density’, ‘biomass’ and ‘community metabolism’ were log_10_-transformed to meet assumptions of normality and ‘species richness’ (overdispersed poisson-distributed data) was modelled on a negative binomial distribution. We additionally explored biodiversity–ecosystem functioning relationships by first testing for linearity of relationships using untransformed data. Once linearity was established, we then tested for the effects of log_10_-transformed ‘species richness’ and its interaction with ‘transformation system’ on ‘energy flux’ for overall data and repeated again for data from separate feeding guilds. In addition, because we suspected that our analyses could be affected by spatial autocorrelation, we calculated Moran’s *I* values for each model’s residuals and tested for spatial autocorrelation using the Moran’s *I* standard deviate^41^ in the ‘spdep’ package in R 3.0.2 (ref. 39). Results from these tests provided no support for the spatial autocorrelation of variation in any of the response variables tested (all Moran’s *I* test results yielded *P*>0.4).

For all GLMM’s, we applied a backward stepwise selection procedure to obtain the model of best fit, based on the Akaike Information Criterion (AIC). In this procedure, we constructed full models that contained all possible predictors and their interactions (‘transformation system’ and ‘feeding guild’ for general community response models; ‘species richness’ and ‘transformation system’ for biodiversity–ecosystem functioning models) and compared these full models and the model of the backward selection procedure to a null, intercept-only model. The model that yielded the lowest AIC score, with a minimum ΔAIC of 2 units, was selected as the model of best fit. All analyses were conducted with the ‘nlme’ and ‘lme4’ packages in R 3.0.2 (ref. 39).

## Author contributions

A.D.B., M.J. and U.B. designed the study; A.D.B., M.J. and S.M. carried out the field and laboratory work; A.D.B. and M.J. prepared and analysed the data; all authors interpreted the results and wrote the paper.

## Additional information

**How to cite this article:** Barnes, A. D. *et al.* Consequences of tropical land use for multitrophic biodiversity and ecosystem functioning. *Nat. Commun.* 5:5351 doi: 10.1038/ncomms6351 (2014).

## Supplementary Material

Supplementary Information Supplementary Figures 1-4, Supplementary Tables 1-5, Supplementary Methods and Supplementary References
