# Supplementary material for: Consequences of tropical land use for multitrophic biodiversity and ecosystem functioning
Source: Nat Commun. 2014 Oct 28;5:5351. doi: 10.1038/ncomms6351 (PMC4220457; doi:10.1038/ncomms6351)
Supplement: Supplementary Information — Supplementary Figures 1-4, Supplementary Tables 1-5, Supplementary Methods and Supplementary References [file ncomms6351-s1.pdf]

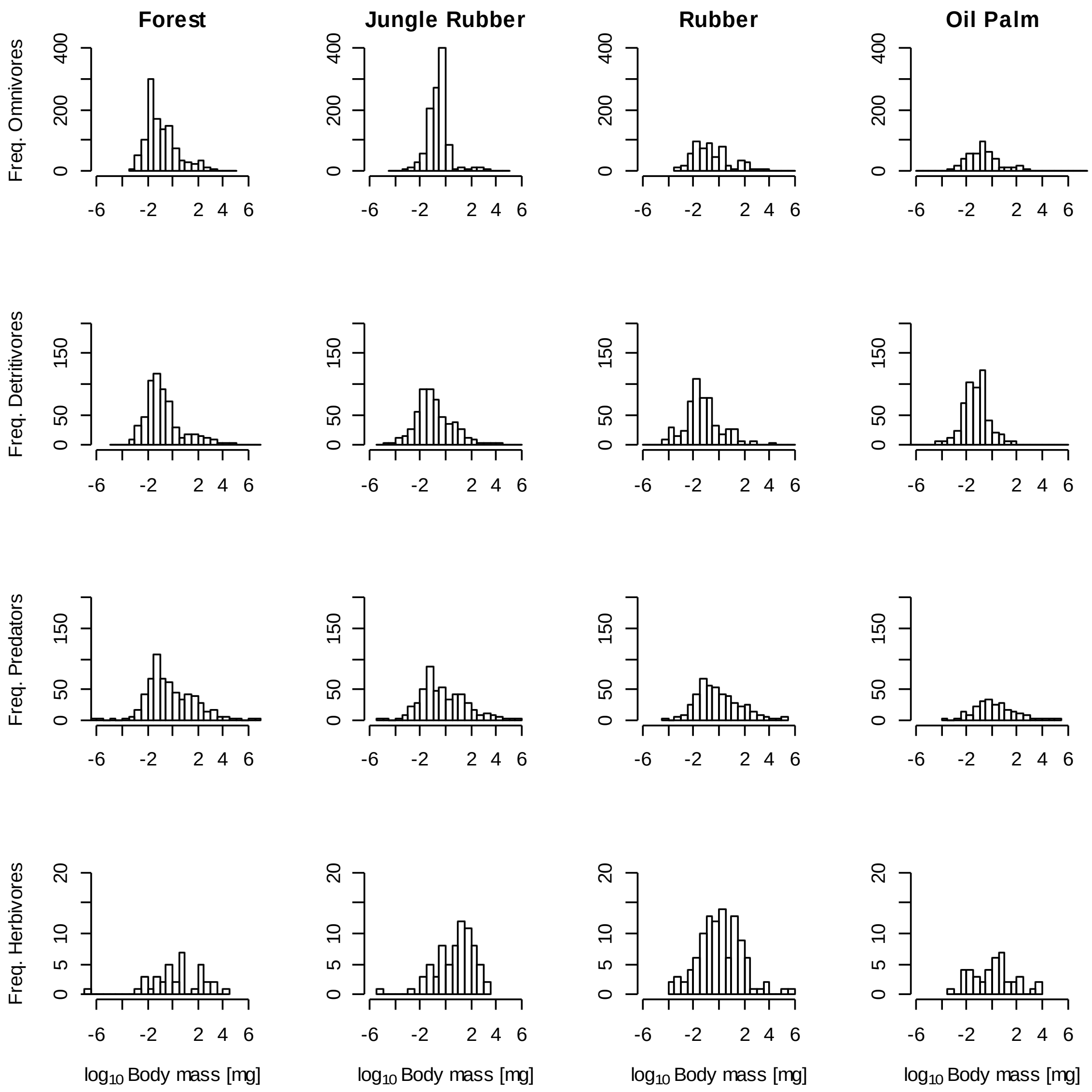

Supplementary Figure 1. Body mass distributions across the four transformation systems for each of the four functional feeding guilds: omnivores (3209 individuals), detritivores (2242 individuals), predators (1766 individuals), and herbivores (255 individuals).

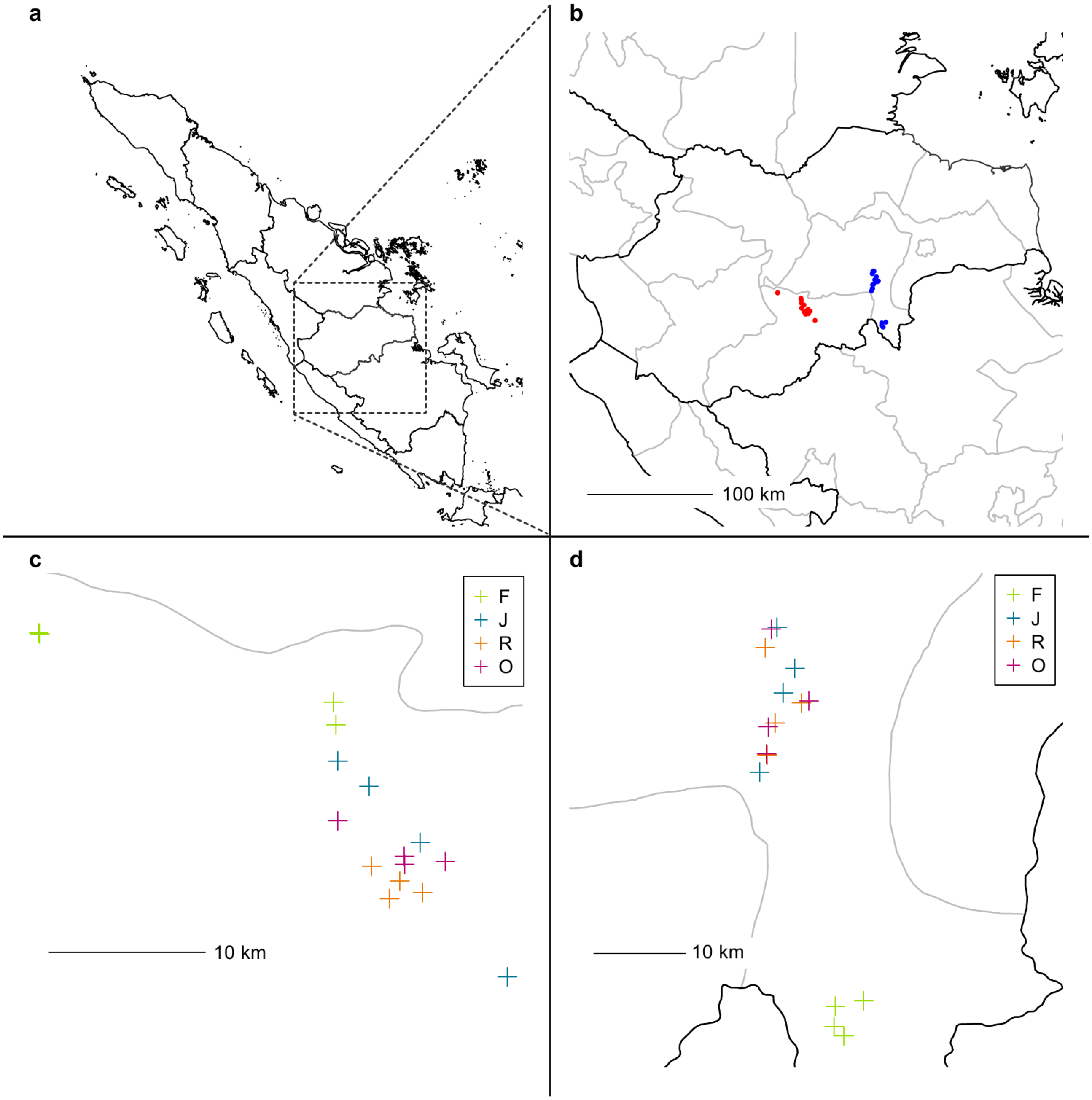

Supplementary Figure 2. Map of the study region with an overview of Sumatra (a) and Jambi Province (b) with red and blue points denoting the 16 sites in Bukit Duabelas landscape and the 16 sites in Harapan landscape, respectively. Additionally, the spatial layout of the sampling sites in Bukit Duabelas landscape (c) and Harapan landscape (d) is represented by coloured crosses for forest (F), jungle rubber (J), rubber (R) and oil palm (O).

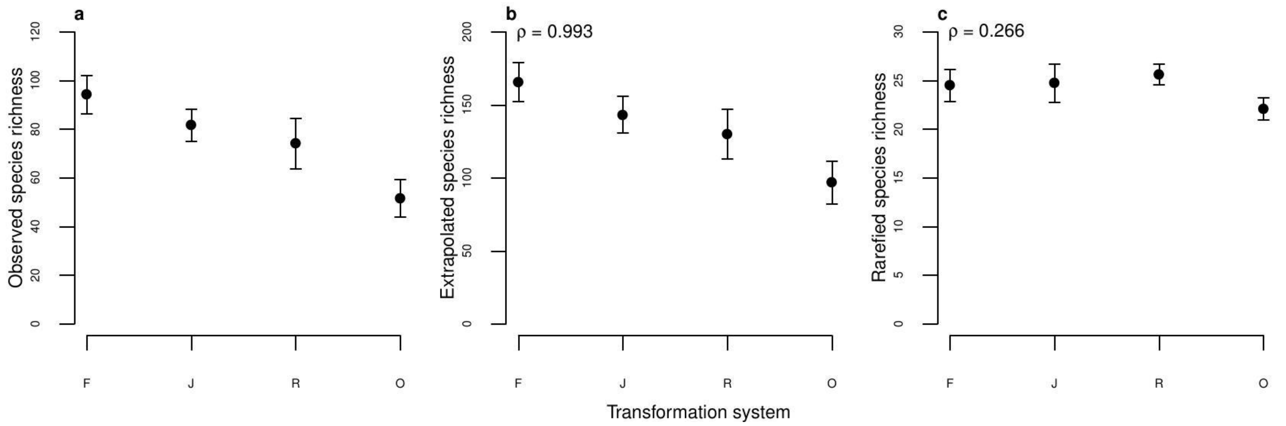

Supplementary Figure 3. Mean (± SE) observed species richness (a), 2nd order jacknife extrapolated species richness (b) and rarefied species richness (c) for the four land-use transformation systems: forest (F), jungle rubber (J), rubber (R) and oil palm (O). ρ-values denote Pearson correlation coefficients between observed species richness and extrapolated (b) and rarefied species richness (c) for the 32 sites (n = 32), respectively.

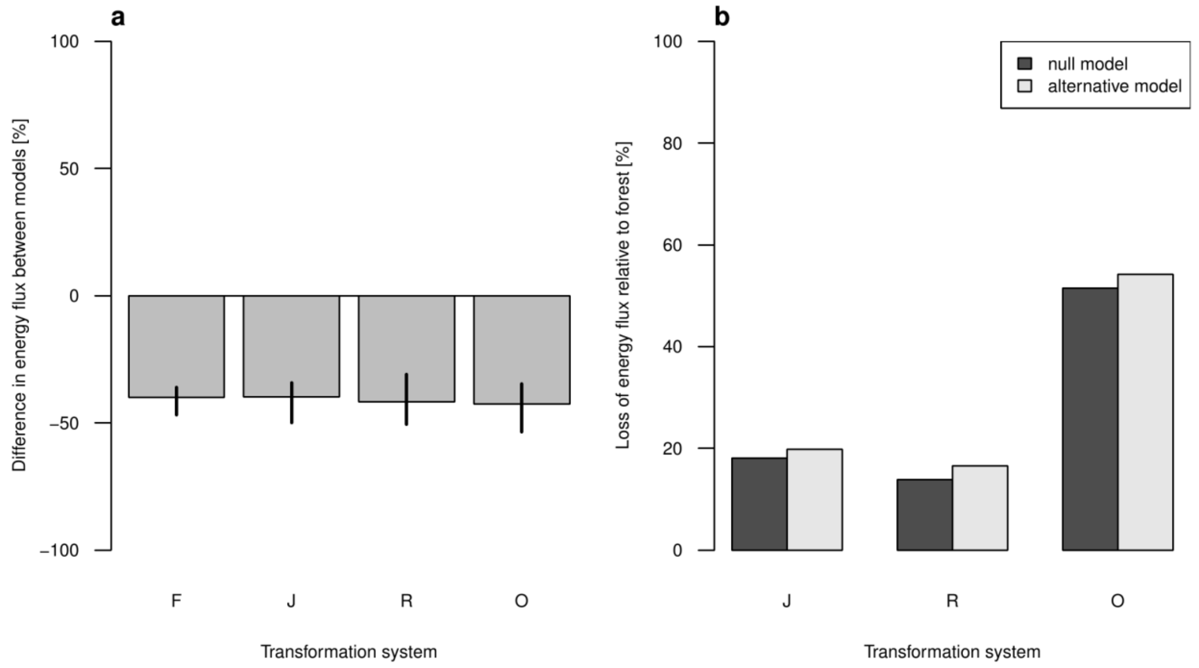

Supplementary Figure 4. (a) Comparative mean percentage change in total energy flux for the four transformation systems between our feeding link assumption null-model (Supplementary Methods) and an alternative flux calculation with omnivores consuming only live plant material and detritus (50% each). Error bars denote upper and lower limits of absolute deviation from the mean. (b) Mean percentage loss in energy flux of the three agriculturally used transformation systems compared to the forest system. Dark grey and light grey bars denote the null model and alternative model calculations, respectively. Transformation system abbreviations are: forest (F), jungle rubber (J), rubber (R) and oil palm (O).

Supplementary Table 1. Summary and ANOVA tables from the best-fit generalized linear mixed effects models as selected by AIC: (a) negative binomial model testing the effects of transformation system (TrSys) and functional feeding guild (FFG) on species richness (SpRichness); (b) gaussian models testing the effects of transformation system (TrSys) and functional feeding guild (FFG) on density, biomass, and community metabolism (CM). Asterisks denote significance levels: * $p < 0.05$; ** $p < 0.01$; *** $p < 0.001$.

(a)

Model	Fixed effects	Estimate	Std. Error	z value	Pr(>|z|)	
SpRichness ~ TrSys * FFG	Intercept	3.088	0.127	24.290	0.000	***
	Jungle rubber	0.054	0.179	0.299	0.766	
	Oil palm	-0.556	0.190	-2.921	0.003	**
	Rubber	-0.275	0.184	-1.491	0.135	
	Omnivores	0.168	0.101	1.668	0.096	
	Herbivores	-1.759	0.194	-9.065	0.000	***
	Predators	0.606	0.092	6.553	0.000	***
	Jungle rubber : Omnivores	-0.399	0.149	-2.685	0.007	**
	Oil palm : Omnivores	-0.087	0.168	-0.520	0.603	
	Rubber : Omnivores	-0.099	0.154	-0.644	0.519	
	Jungle rubber : Herbivores	0.346	0.254	1.361	0.174	
	Oil palm : Herbivores	0.641	0.274	2.337	0.019	*
	Rubber : Herbivores	0.953	0.246	3.876	0.000	***
	Jungle rubber : Predators	-0.253	0.132	-1.912	0.056	
	Oil palm : Predators	-0.235	0.156	-1.510	0.131	
	Rubber : Predators	-0.078	0.141	-0.556	0.578	

(b)

Model	Fixed effects	numDF	denDF	F-value	Pr(>|z|)	
Density ~ TrSys * FFG	TrSys	3	27	0.363	0.780	
	FFG	3	84	77.611	0.000	***
	TrSys : FFG	9	84	3.432	0.001	**
Biomass ~ TrSys + FFG	TrSys	3	27	3.570	0.027	*
	FFG	3	93	38.759	0.000	***
CM ~ TrSys + FFG	TrSys	3	27	3.456	0.030	*
	FFG	3	93	64.825	0.000	***

Supplementary Table 2. Energy flux and fresh biomass values for the four functional feeding guilds (FFG) and four transformation systems. Energy flux is expressed as kg fresh mass [ha^{-1} yr^{-1}] using a conversion factor[1]: 1 kg wet mass = 7 *10^6 J.

FFG	Transformation system	Energy flux [kg ha^{-1} yr^{-1}]	Biomass [kg ha^{-1}]
Omnivore	Forest	61.900	0.629
Omnivore	Jungle rubber	52.313	0.494
Omnivore	Rubber	55.880	0.751
Omnivore	Oil palm	32.531	0.766
Detritivore	Forest	200.187	1.039
Detritivore	Jungle rubber	160.165	0.558
Detritivore	Rubber	164.194	0.504
Detritivore	Oil palm	94.440	0.352
Predator	Forest	66.816	1.664
Predator	Jungle rubber	53.248	0.976
Predator	Rubber	55.454	0.954
Predator	Oil palm	30.697	0.424
Herbivore	Forest	87.537	0.093
Herbivore	Jungle	75.389	0.139
Herbivore	Rubber	83.288	0.319
Herbivore	Oil palm	44.316	0.076

Supplementary Table 3. ANOVA tables from the generalized linear mixed effects models testing the effects of transformation system (TrSys), species richness (SpRichness), and their interaction on energy flux (EF) for the total community data set and also separated into functional feeding guilds (FFG). All models displayed are those that were selected as the best-fit model from the stepwise AIC selection procedure. Asterisks denote significance levels: * p < 0.05; ** p < 0.01; *** p < 0.001.

Model	Fixed effects	numDF	denDF	F-value	Pr(>|z|)	
**Total Community**<br>EF ~ TrSys * SpRichness	TrSys	3	23	5.226	0.007	**
	SpRichness	1	23	4.965	0.036	*
	TrSys : SpRichness	3	23	4.637	0.011	*
**Omnivores**<br>EF ~ SpRichness	SpRichness	1	29	42.842	0.000	***
**Detritivores**<br>EF ~ TrSys + SpRichness	TrSys	3	26	3.103	0.044	*
	SpRichness	1	26	22.285	0.000	***
**Predators**<br>EF ~ TrSys * SpRichness	TrSys	3	23	5.507	0.005	**
	SpRichness	1	23	5.813	0.024	*
	TrSys : SpRichness	3	23	4.618	0.011	*
**Herbivores**<br>EF ~ TrSys + SpRichness	TrSys	3	26	5.944	0.003	**
	SpRichness	1	26	9.436	0.005	**

Supplementary Table 4a. Length-mass regression parameters for calculation of individual body masses from measured body lengths. For damaged individuals where body length could not be measured (66 of 7472 individuals), body mass was substituted by species median body mass or order median body mass (for species with single individuals). 'Taxon', 'Group' and 'Further grouping' specify which animals the presented regression has been used for in this study. Regressions were available from the literature that estimate both dry and fresh mass ('Mass type') for different taxa. Supplementary table 4b presents the dry mass-fresh mass conversions, used to convert all estimated body masses to fresh mass. The equations and regression parameters, 'a' and 'b', are presented, as well as the size range the regressions were calculated from ('Min' and 'Max'). All regressions were taken from the literature ('Reference'), with different specific definitions of how body length was measured ('Details of body length measurement') and specificity of the given regression ('Regression specificity').

Taxon	Group	Further grouping	Mass type	Equation M[mg], L[mm]	a	b	Min (mm)	Max (mm)	Reference	Details of body length measurement	Regression specificity
Annelida	All		ash free dry mass	M = 1000*exp(a + b * log(L))	-11.8423	2.3225			(Hale, Reich & Frelich, 2004)	Total length	General Lumbricidae
Araneae	Araneae < 2.5 mm		Fresh mass	M = exp(a + b * log(L))	-1.958	2.746	0.56	2.5	(Höfer & Ott, 2009)	edge of prosoma (without chelicerae) to edge of opisthosoma (excl spinnerets)	Group specific
Araneae	hunting		Fresh mass	M = exp(a + b * log(L))	-2.108	3.017	0.67	36	(Höfer & Ott, 2009)	edge of prosoma (without chelicerae) to edge of opisthosoma (excl spinnerets)	Group specific
Araneae	web-building		Fresh mass	M = exp(a + b * log(L))	-1.784	2.255	0.56	10.67	(Höfer & Ott, 2009)	edge of prosoma (without chelicerae) to edge of opisthosoma (excl spinnerets)	Group specific
Araneae	spiders random		fresh mass	M = exp(a + b * log(L))	-1.844	2.711	1.8	21.5	(Edwards, 1996)	clypeus to tip of spinnerets	Group specific
Araneae	Anapidae		Fresh mass	M = exp(a + b * log(L))	-1.784	2.255	0.56	10.67	(Höfer & Ott, 2009)	edge of prosoma (without chelicerae) to edge of opisthosoma (excl spinnerets)	inferred, web-building
Araneae	Araneidae		Fresh mass	M = exp(a + b * log(L))	-1.923	2.923	2.10	21.20	(Edwards, 1996)	clypeus to tip of spinnerets	Group specific
Araneae	Barychelidae		Fresh mass	M = exp(a + b * log(L))	-2.108	3.017	0.67	36	(Höfer & Ott, 2009)	edge of prosoma (without chelicerae) to edge of opisthosoma (excl spinnerets)	inferred, hunting spiders
Araneae	Clubionidae		Fresh mass	M = exp(a + b * log(L))	-2.156	2.653	2.5	9	(Edwards, 1996)	clypeus to tip of spinnerets	Group specific
Araneae	Corinnidae		Fresh mass	M = exp(a + b * log(L))	-2.108	3.017	0.67	36	(Höfer & Ott, 2009)	edge of prosoma (without chelicerae) to edge of opisthosoma (excl spinnerets)	inferred, hunting spiders
Araneae	Ctenidae		Fresh mass	M = exp(a + b * log(L))	-1.758	2.894	1.3	36	(Höfer & Ott, 2009)	edge of prosoma (without chelicerae) to edge of opisthosoma (excl spinnerets)	Group specific
Araneae	Deinopidae		fresh mass	M = exp(a + b * log(L))	-1.844	2.711	1.8	21.5	(Edwards, 1996)	clypeus to tip of spinnerets	inferred, spiders random sample
Araneae	Gnaphosidae		Fresh mass	M = exp(a + b * log(L))	-2.83	3.055	3	13.1	(Edwards, 1996)	clypeus to tip of spinnerets	Group specific

Araneae	Hexathelidae		Fresh mass	M = exp(a + b * log(L))	-2.108	3.017	0.67	36	(Höfer & Ott, 2009)	edge of prosoma (without chelicerae) to edge of opisthosoma (excl spinnerets)	inferred, hunting spiders
Araneae	Lamponidae		Fresh mass	M = exp(a + b * log(L))	-2.83	3.055	3	13.1	(Edwards, 1996)	clypeus to tip of spinnerets	inferred, Gnaphosidae
Araneae	Linyphiidae		Fresh mass	M = exp(a + b * log(L))	-1.892	2.754	1.5	5.5	(Edwards, 1996)	clypeus to tip of spinnerets	Group specific
Araneae	Lycosidae		Fresh mass	M = exp(a + b * log(L))	-2.043	2.842	2	23.5	(Edwards, 1996)	clypeus to tip of spinnerets	Group specific
Araneae	Micropholcommatidae		Fresh mass	M = exp(a + b * log(L))	-1.784	2.255	0.56	10.67	(Höfer & Ott, 2009)	edge of prosoma (without chelicerae) to edge of opisthosoma (excl spinnerets)	inferred, web-building
Araneae	Miturgidae		Fresh mass	M = exp(a + b * log(L))	-2.83	3.055	3	13.1	(Edwards, 1996)	clypeus to tip of spinnerets	inferred, Gnaphosidae
Araneae	Mysmenidae		Fresh mass	M = exp(a + b * log(L))	-1.784	2.255	0.56	10.67	(Höfer & Ott, 2009)	edge of prosoma (without chelicerae) to edge of opisthosoma (excl spinnerets)	inferred, web-building
Araneae	Nemesiidae		Fresh mass	M = exp(a + b * log(L))	-2.108	3.017	0.67	36	(Höfer & Ott, 2009)	edge of prosoma (without chelicerae) to edge of opisthosoma (excl spinnerets)	inferred, hunting spiders
Araneae	Nephilidae		Fresh mass	M = exp(a + b * log(L))	-1.784	2.255	0.56	10.67	(Höfer & Ott, 2009)	edge of prosoma (without chelicerae) to edge of opisthosoma (excl spinnerets)	inferred, web-building
Araneae	Ochyroceratidae		Fresh mass	M = exp(a + b * log(L))	-1.784	2.255	0.56	10.67	(Höfer & Ott, 2009)	edge of prosoma (without chelicerae) to edge of opisthosoma (excl spinnerets)	inferred, web-building
Araneae	Oonopidae		Fresh mass	M = exp(a + b * log(L))	-2.039	2.666	0.67	2.5	(Höfer & Ott, 2009)	edge of prosoma (without chelicerae) to edge of opisthosoma (excl spinnerets)	Group specific
Araneae	Oxyopidae		Fresh mass	M = exp(a + b * log(L))	-2.108	3.017	0.67	36	(Höfer & Ott, 2009)	edge of prosoma (without chelicerae) to edge of opisthosoma (excl spinnerets)	inferred, hunting spiders
Araneae	Palpimanidae		Fresh mass	M = exp(a + b * log(L))	-2.108	3.017	0.67	36	(Höfer & Ott, 2009)	edge of prosoma (without chelicerae) to edge of opisthosoma (excl spinnerets)	inferred, hunting spiders
Araneae	Pararchaeidae		Fresh mass	M = exp(a + b * log(L))	-2.108	3.017	0.67	36	(Höfer & Ott, 2009)	edge of prosoma (without chelicerae) to edge of opisthosoma (excl spinnerets)	inferred, hunting spiders
Araneae	Philodromidae		Fresh mass	M = exp(a + b * log(L))	-1.985	2.940	2.50	8.60	(Edwards, 1996)	clypeus to tip of spinnerets	Group specific
Araneae	Pholcidae		Fresh mass	M = exp(a + b * log(L))	-1.784	2.255	0.56	10.67	(Höfer & Ott, 2009)	edge of prosoma (without chelicerae) to edge of opisthosoma (excl spinnerets)	inferred, web-building
Araneae	Prodidomidae		Fresh mass	M = exp(a + b * log(L))	-2.83	3.055	3	13.1	(Edwards, 1996)	clypeus to tip of spinnerets	inferred, Gnaphosidae
Araneae	Salticidae		Fresh mass	M = exp(a + b * log(L))	-2.184	2.901	4.00	13.00	(Edwards, 1996)	clypeus to tip of spinnerets	Group specific
Araneae	Scytodidae		Fresh mass	M = exp(a + b * log(L))	-2.108	3.017	0.67	36	(Höfer & Ott, 2009)	edge of prosoma (without chelicerae) to edge of opisthosoma (excl spinnerets)	inferred, hunting spiders
Araneae	Segestriidae		Fresh mass	M = exp(a + b * log(L))	-2.108	3.017	0.67	36	(Höfer & Ott, 2009)	edge of prosoma (without chelicerae) to edge of opisthosoma (excl spinnerets)	inferred, hunting spiders

Araneae	Sparassidae		Fresh mass	M = exp(a + b * log(L))	-2.108	3.017	0.67	36	(Höfer & Ott, 2009)	edge of prosoma (without chelicerae) to edge of opisthosoma (excl spinnerets)	inferred, hunting spiders
Araneae	Stenochilidae		Fresh mass	M = exp(a + b * log(L))	-2.108	3.017	0.67	36	(Höfer & Ott, 2009)	edge of prosoma (without chelicerae) to edge of opisthosoma (excl spinnerets)	inferred, hunting spiders
Araneae	Symphytognathidae		Fresh mass	M = exp(a + b * log(L))	-1.784	2.255	0.56	10.67	(Höfer & Ott, 2009)	edge of prosoma (without chelicerae) to edge of opisthosoma (excl spinnerets)	inferred, web-building
Araneae	Telemidae		Fresh mass	M = exp(a + b * log(L))	-1.784	2.255	0.56	10.67	(Höfer & Ott, 2009)	edge of prosoma (without chelicerae) to edge of opisthosoma (excl spinnerets)	inferred, web-building
Araneae	Tetrablemmidae		Fresh mass	M = exp(a + b * log(L))	-2.039	2.666	0.67	2.5	(Höfer & Ott, 2009)	edge of prosoma (without chelicerae) to edge of opisthosoma (excl spinnerets)	inferred, Oonopidae
Araneae	Tetragnathidae		Fresh mass	M = exp(a + b * log(L))	-2.615	2.574	3.50	9.00	(Edwards, 1996)	clypeus to tip of spinnerets	Group specific
Araneae	Theridiidae		Fresh mass	M = exp(a + b * log(L))	-1.577	2.907	1.50	7.50	(Edwards, 1996)	clypeus to tip of spinnerets	Group specific
Araneae	Theridiosomatidae		Fresh mass	M = exp(a + b * log(L))	-1.784	2.255	0.56	10.67	(Höfer & Ott, 2009)	edge of prosoma (without chelicerae) to edge of opisthosoma (excl spinnerets)	inferred, web-building
Araneae	Thomisidae		Fresh mass	M = exp(a + b * log(L))	-1.644	2.973	1.80	8.00	(Edwards, 1996)	clypeus to tip of spinnerets	Group specific
Araneae	Uloboridae		Fresh mass	M = exp(a + b * log(L))	-1.784	2.255	0.56	10.67	(Höfer & Ott, 2009)	edge of prosoma (without chelicerae) to edge of opisthosoma (excl spinnerets)	inferred, web-building
Araneae	Unidentifiable < 1.8		Fresh mass	M = exp(a + b * log(L))	-1.958	2.746	0.56	2.5	(Höfer & Ott, 2009)	edge of prosoma (without chelicerae) to edge of opisthosoma (excl spinnerets)	inferred, Araneae < 2.5 mm
Araneae	Unidentifiable > 1.8		fresh mass	M = exp(a + b * log(L))	-1.844	2.711	1.8	21.5	(Edwards, 1996)	clypeus to tip of spinnerets	inferred, spiders random sample
Araneae	Zodariidae		Fresh mass	M = exp(a + b * log(L))	-2.108	3.017	0.67	36	(Höfer & Ott, 2009)	edge of prosoma (without chelicerae) to edge of opisthosoma (excl spinnerets)	inferred, hunting spiders
Archaeognatha	All		Dry mass	M = exp(a + b * log(L))	-3.628	2.494	2.13	54.51	(Sample, Cooper, Greer, & Whitmore, 1993)	From frons to tip of abdomen excluding appendages	inferred, all insect taxa
Blattodea	Blaberidae		Dry mass	M = exp(a + b * log(L))	-3.980	2.760	2.20	14.00	(Wardhaugh, 2013)	front of labrum to tip of abdomen (excl. cerci or ovipositors) or tip of elytra (longest)	inferred, Blattodea
Blattodea	Blattellidae		Dry mass	M = exp(a + b * log(L))	-3.980	2.760	2.20	14.00	(Wardhaugh, 2013)	front of labrum to tip of abdomen (excl. cerci or ovipositors) or tip of elytra (longest)	inferred, Blattodea
Blattodea	Blattidae		Dry mass	M = exp(a + b * log(L))	-3.980	2.760	2.20	14.00	(Wardhaugh, 2013)	front of labrum to tip of abdomen (excl. cerci or ovipositors) or tip of elytra (longest)	inferred, Blattodea
Blattodea	Unidentifiable		Dry mass	M = exp(a + b * log(L))	-3.980	2.760	2.20	14.00	(Wardhaugh, 2013)	front of labrum to tip of abdomen (excl. cerci or ovipositors) or tip of elytra (longest)	inferred, Blattodea
Chilopoda	Ballophilidae		Dry mass	M = exp(a + b * log(L))	-4.049	2.18	4	47	(Gowing & Recher, 1984)	not mentioned	inferred, Chilopoda
Chilopoda	Cryptopidae		Dry mass	M = exp(a + b * log(L))	-4.049	2.18	4	47	(Gowing & Recher, 1984)	not mentioned	inferred, Chilopoda

Chilopoda	Henicopidae		Dry mass	M = exp(a + b * log(L))	-4.049	2.18	4	47	(Gowing & Recher, 1984)	not mentioned	inferred, Chilopoda
Chilopoda	Lithobiomorpha		Dry mass	M = exp(a + b * log(L))	-4.049	2.18	4	47	(Gowing & Recher, 1984)	not mentioned	inferred, Chilopoda
Chilopoda	Mecistocephalidae		Dry mass	M = exp(a + b * log(L))	-4.049	2.18	4	47	(Gowing & Recher, 1984)	not mentioned	inferred, Chilopoda
Chilopoda	Scolopendridae		Dry mass	M = exp(a + b * log(L))	-4.049	2.18	4	47	(Gowing & Recher, 1984)	not mentioned	inferred, Chilopoda
Chilopoda	Unidentifiable		Dry mass	M = exp(a + b * log(L))	-4.049	2.18	4	47	(Gowing & Recher, 1984)	not mentioned	inferred, Chilopoda
Coleoptera	Anobiidae		Dry mass	M = exp(a + b * log(L))	-3.247	2.492	3.34	34.82	(Sample, Cooper, Greer, & Whitmore, 1993)	From frons to tip of abdomen excluding appendages	inferred, Coleoptera
Coleoptera	Anthicidae		Dry mass	M = exp(a + b * log(L))	-3.247	2.492	3.34	34.82	(Sample, Cooper, Greer, & Whitmore, 1993)	From frons to tip of abdomen excluding appendages	inferred, Coleoptera
Coleoptera	Bostrichidae		Dry mass	M = exp(a + b * log(L))	-3.247	2.492	3.34	34.82	(Sample, Cooper, Greer, & Whitmore, 1993)	From frons to tip of abdomen excluding appendages	inferred, Coleoptera
Coleoptera	Byrrhidae		Dry mass	M = exp(a + b * log(L))	-3.247	2.492	3.34	34.82	(Sample, Cooper, Greer, & Whitmore, 1993)	From frons to tip of abdomen excluding appendages	inferred, Coleoptera
Coleoptera	Carabidae		Dry mass	M = a * L^b	0.0237	2.7054	2.88	24	(Lang, Krooss & Stumpf, 1997)	Measured from anterior tip of head to posterior of abdomen excluding any appendages	Group specific
Coleoptera	Cerylonidae		Dry mass	M = exp(a + b * log(L))	-3.247	2.492	3.34	34.82	(Sample, Cooper, Greer, & Whitmore, 1993)	From frons to tip of abdomen excluding appendages	inferred, Coleoptera
Coleoptera	Chelonariidae		Dry mass	M = exp(a + b * log(L))	-3.247	2.492	3.34	34.82	(Sample, Cooper, Greer, & Whitmore, 1993)	From frons to tip of abdomen excluding appendages	inferred, Coleoptera
Coleoptera	Chrysomelidae		Dry mass	M = exp(a + b * log(L))	-2.427	2.171	3.34	7.84	(Sample, Cooper, Greer, & Whitmore, 1993)	From frons to tip of abdomen excluding appendages	Group specific
Coleoptera	Ciidae		Dry mass	M = exp(a + b * log(L))	-3.247	2.492	3.34	34.82	(Sample, Cooper, Greer, & Whitmore, 1993)	From frons to tip of abdomen excluding appendages	inferred, Coleoptera
Coleoptera	Cleridae		Dry mass	M = exp(a + b * log(L))	-3.247	2.492	3.34	34.82	(Sample, Cooper, Greer, & Whitmore, 1993)	From frons to tip of abdomen excluding appendages	inferred, Coleoptera
Coleoptera	Coccinellidae		Dry mass	M = exp(a + b * log(L))	-3.247	2.492	3.34	34.82	(Sample, Cooper, Greer, & Whitmore, 1993)	From frons to tip of abdomen excluding appendages	inferred, Coleoptera
Coleoptera	Colydiidae		Dry mass	M = exp(a + b * log(L))	-3.247	2.492	3.34	34.82	(Sample, Cooper, Greer, & Whitmore, 1993)	From frons to tip of abdomen excluding appendages	inferred, Coleoptera
Coleoptera	Curculionidae		Dry mass	M = exp(a + b * log(L))	-3.247	2.492	3.34	34.82	(Sample, Cooper, Greer, & Whitmore, 1993)	From frons to tip of abdomen excluding appendages	inferred, Coleoptera
Coleoptera	Dermestidae		Dry mass	M = exp(a + b * log(L))	-3.247	2.492	3.34	34.82	(Sample, Cooper, Greer, & Whitmore, 1993)	From frons to tip of abdomen excluding appendages	inferred, Coleoptera
Coleoptera	Discolomidae		Dry mass	M = exp(a + b * log(L))	-3.247	2.492	3.34	34.82	(Sample, Cooper, Greer, & Whitmore, 1993)	From frons to tip of abdomen excluding appendages	inferred, Coleoptera

Coleoptera	Elateridae		Dry mass	M = a * L^b	0.0138	2.595	1.65	10.3	(Gruner, 2003)	tip of abdomen to end of head or carapace, excl. any appendages	inferred, slender beetles
Coleoptera	Endomychidae		Dry mass	M = exp(a + b * log(L))	-3.247	2.492	3.34	34.82	(Sample, Cooper, Greer, & Whitmore, 1993)	From frons to tip of abdomen excluding appendages	inferred, Coleoptera
Coleoptera	Histeridae		Dry mass	M = exp(a + b * log(L))	-3.247	2.492	3.34	34.82	(Sample, Cooper, Greer, & Whitmore, 1993)	From frons to tip of abdomen excluding appendages	inferred, Coleoptera
Coleoptera	Hydrophilidae		Dry mass	M = exp(a + b * log(L))	-3.247	2.492	3.34	34.82	(Sample, Cooper, Greer, & Whitmore, 1993)	From frons to tip of abdomen excluding appendages	inferred, Coleoptera
Coleoptera	Languriidae		Dry mass	M = exp(a + b * log(L))	-3.247	2.492	3.34	34.82	(Sample, Cooper, Greer, & Whitmore, 1993)	From frons to tip of abdomen excluding appendages	inferred, Coleoptera
Coleoptera	Larvae		Dry mass	M = a * L^b	0.0035	2.4033	1.5	25.27	(Lang, Krooss, & Stumpf, 1997)	Measured from anterior tip of head to posterior of abdomen excluding any appendages	inferred, Coleoptera
Coleoptera	Leiodidae		Dry mass	M = exp(a + b * log(L))	-3.247	2.492	3.34	34.82	(Sample, Cooper, Greer, & Whitmore, 1993)	From frons to tip of abdomen excluding appendages	inferred, Coleoptera
Coleoptera	Lucanidae		Dry mass	M = exp(a + b * log(L))	-3.247	2.492	3.34	34.82	(Sample, Cooper, Greer, & Whitmore, 1993)	From frons to tip of abdomen excluding appendages	inferred, Coleoptera
Coleoptera	Melyridae		Dry mass	M = exp(a + b * log(L))	-3.247	2.492	3.34	34.82	(Sample, Cooper, Greer, & Whitmore, 1993)	From frons to tip of abdomen excluding appendages	inferred, Coleoptera
Coleoptera	Mordellidae		Dry mass	M = exp(a + b * log(L))	-3.247	2.492	3.34	34.82	(Sample, Cooper, Greer, & Whitmore, 1993)	From frons to tip of abdomen excluding appendages	inferred, Coleoptera
Coleoptera	Mycetophagidae		Dry mass	M = exp(a + b * log(L))	-3.247	2.492	3.34	34.82	(Sample, Cooper, Greer, & Whitmore, 1993)	From frons to tip of abdomen excluding appendages	inferred, Coleoptera
Coleoptera	Pselaphidae		Dry mass	M = exp(a + b * log(L))	-3.247	2.492	3.34	34.82	(Sample, Cooper, Greer, & Whitmore, 1993)	From frons to tip of abdomen excluding appendages	inferred, Coleoptera
Coleoptera	Ptiliidae		Dry mass	M = exp(a + b * log(L))	-3.247	2.492	3.34	34.82	(Sample, Cooper, Greer, & Whitmore, 1993)	From frons to tip of abdomen excluding appendages	inferred, Coleoptera
Coleoptera	Scarabaeidae		Dry mass	M = exp(a + b * log(L))	-2.448	2.494	4.24	24.79	(Sample, Cooper, Greer, & Whitmore, 1993)	From frons to tip of abdomen excluding appendages	Group specific
Coleoptera	Scydmaenidae		Dry mass	M = exp(a + b * log(L))	-3.247	2.492	3.34	34.82	(Sample, Cooper, Greer, & Whitmore, 1993)	From frons to tip of abdomen excluding appendages	inferred, Coleoptera
Coleoptera	Silvanidae		Dry mass	M = a * L^b	0.0138	2.595	1.65	10.3	(Gruner, 2003)	tip of abdomen to end of head or carapace, excl. any appendages	inferred, slender beetles
Coleoptera	Staphylinidae		Dry mass	M = a * L^b	0.0134	2.26	2.2	13.6	(Lang, Krooss, & Stumpf, 1997)	Measured from anterior tip of head to posterior of abdomen excluding any appendages	Group specific
Coleoptera	Tenebrionidae		Dry mass	M = exp(a + b * log(L))	-0.043	1.2	5.65	13.39	(Sample, Cooper, Greer, & Whitmore, 1993)	From frons to tip of abdomen excluding appendages	Group specific
Coleoptera	Throscidae		Dry mass	M = exp(a + b * log(L))	-3.247	2.492	3.34	34.82	(Sample, Cooper, Greer, & Whitmore, 1993)	From frons to tip of abdomen excluding appendages	inferred, Coleoptera
Coleoptera	Trogossitidae		Dry mass	M = exp(a + b * log(L))	-3.247	2.492	3.34	34.82	(Sample, Cooper, Greer, & Whitmore, 1993)	From frons to tip of abdomen excluding appendages	inferred, Coleoptera

Coleoptera	Unidentifiable		Dry mass	M = exp(a + b * log(L))	-3.247	2.492	3.34	34.82	(Sample, Cooper, Greer, & Whitmore, 1993)	From frons to tip of abdomen excluding appendages	inferred, Coleoptera
Coleoptera	Zopheridae		Dry mass	M = exp(a + b * log(L))	-3.247	2.492	3.34	34.82	(Sample, Cooper, Greer, & Whitmore, 1993)	From frons to tip of abdomen excluding appendages	inferred, Coleoptera
Dermaptera	Anisolabididae		Dry mass	M = exp(a + b * log(L))	-3.628	2.494	2.13	54.51	(Sample, Cooper, Greer, & Whitmore, 1993)	From frons to tip of abdomen excluding appendages	inferred, all insect taxa
Dermaptera	Forficulidae		Dry mass	M = exp(a + b * log(L))	-3.628	2.494	2.13	54.51	(Sample, Cooper, Greer, & Whitmore, 1993)	From frons to tip of abdomen excluding appendages	inferred, all insect taxa
Diplopoda	Chordeumatida		Dry mass	M = exp(a + b * log(L))	-4.591	2.543	11.0	47.0	(Gowing & Recher, 1984)	not mentioned	inferred, Diplopoda
Diplopoda	Glomerida		Dry mass	M = exp(a + b * log(L))	-4.591	2.543	11.0	47.0	(Gowing & Recher, 1984)	not mentioned	inferred, Diplopoda
Diplopoda	Polidesmatidae		Dry mass	M = exp(a + b * log(L))	-4.591	2.543	11.0	47.0	(Gowing & Recher, 1984)	not mentioned	inferred, Diplopoda
Diplopoda	Polydesmatida		Dry mass	M = exp(a + b * log(L))	-4.591	2.543	11.0	47.0	(Gowing & Recher, 1984)	not mentioned	inferred, Diplopoda
Diplopoda	Polydesmida		Dry mass	M = exp(a + b * log(L))	-4.591	2.543	11.0	47.0	(Gowing & Recher, 1984)	not mentioned	inferred, Diplopoda
Diplopoda	Polydesmidae		Dry mass	M = exp(a + b * log(L))	-4.591	2.543	11.0	47.0	(Gowing & Recher, 1984)	not mentioned	inferred, Diplopoda
Diplopoda	Polyxenida		Dry mass	M = exp(a + b * log(L))	-4.591	2.543	11.0	47.0	(Gowing & Recher, 1984)	not mentioned	inferred, Diplopoda
Diplopoda	Siphonophorida		Dry mass	M = exp(a + b * log(L))	-4.591	2.543	11.0	47.0	(Gowing & Recher, 1984)	not mentioned	inferred, Diplopoda
Diplopoda	Spirobolida		Dry mass	M = exp(a + b * log(L))	-4.591	2.543	11.0	47.0	(Gowing & Recher, 1984)	not mentioned	inferred, Diplopoda
Diplura	Heterojapygidae		Dry mass	M = a * (L)^b	0.034	2.191	0.9	17.6	(Gruner, 2003)	tip of abdomen to end of head or carapace, excl. any appendages	inferred, general arthropod
Diptera	Larvae		Dry mass	M = a * (L)^b	0.029	1.73	1.7	16.65	(Gruner, 2003)	tip of abdomen to end of head or carapace, excl. any appendages	inferred, holomet. Larvae
Diptera	Adults		Dry mass	M = a * (L)^b	0.0153	2.573	1.75	8.6	(Gruner, 2003)	tip of abdomen to end of head or carapace, excl. any appendages	Group specific, Diptera adult
Diptera	Agromyzidae	Larvae	Dry mass	M = a * (L)^b	0.029	1.73	1.7	16.65	(Gruner, 2003)	tip of abdomen to end of head or carapace, excl. any appendages	inferred, holomet. Larvae
Diptera	Cecidomyiidae	A / L	Dry mass	M = a * (L)^b	0.035	2.173	0.9	17.6	(Gruner, 2003)	tip of abdomen to end of head or carapace, excl. any appendages	inferred, all insect taxa
Diptera	Ceratopogonidae	Larvae	Dry mass	M = a * (L)^b	0.029	1.73	1.7	16.65	(Gruner, 2003)	tip of abdomen to end of head or carapace, excl. any appendages	inferred, holomet. Larvae
Diptera	Chironomidae	Larvae	Dry mass	M = a * (L)^b	0.029	1.73	1.7	16.65	(Gruner, 2003)	tip of abdomen to end of head or carapace, excl. any appendages	inferred, holomet. Larvae

Diptera	Drosophilidae	Larvae	Dry mass	M = a * (L)^b	0.029	1.73	1.7	16.65	(Gruner, 2003)	tip of abdomen to end of head or carapace, excl. any appendages	inferred, holomet. Larvae
Diptera	Muscidae	Adult	Dry mass	M = a * (L)^b	0.0153	2.573	1.75	8.6	(Gruner, 2003)	tip of abdomen to end of head or carapace, excl. any appendages	Group specific, Diptera adult
Diptera	Mycetophilidae	Larvae	Dry mass	M = a * (L)^b	0.029	1.73	1.7	16.65	(Gruner, 2003)	tip of abdomen to end of head or carapace, excl. any appendages	inferred, holomet. Larvae
Diptera	Phoridae	Adult	Dry mass	M = a * (L)^b	0.0153	2.573	1.75	8.6	(Gruner, 2003)	tip of abdomen to end of head or carapace, excl. any appendages	Group specific, Diptera adult
Diptera	Pipunculidae	Larvae	Dry mass	M = a * (L)^b	0.029	1.73	1.7	16.65	(Gruner, 2003)	tip of abdomen to end of head or carapace, excl. any appendages	inferred, holomet. Larvae
Diptera	Sciaridae	Adult	Dry mass	M = a * (L)^b	0.0153	2.573	1.75	8.6	(Gruner, 2003)	tip of abdomen to end of head or carapace, excl. any appendages	Group specific, Diptera adult
Diptera	Simuliidae	Adult	Dry mass	M = a * (L)^b	0.0153	2.573	1.75	8.6	(Gruner, 2003)	tip of abdomen to end of head or carapace, excl. any appendages	Group specific, Diptera adult
Diptera	Syrphidae	Larvae	Dry mass	M = a * (L)^b	0.029	1.73	1.7	16.65	(Gruner, 2003)	tip of abdomen to end of head or carapace, excl. any appendages	inferred, holomet. Larvae
Diptera	Tachinidae	Adult	Dry mass	M = a * (L)^b	0.0153	2.573	1.75	8.6	(Gruner, 2003)	tip of abdomen to end of head or carapace, excl. any appendages	Group specific, Diptera adult
Diptera	Tephritidae	Larvae	Dry mass	M = a * (L)^b	0.029	1.73	1.7	16.65	(Gruner, 2003)	tip of abdomen to end of head or carapace, excl. any appendages	inferred, holomet. Larvae
Diptera	Thaumaleidae	A / L	Dry mass	M = a * (L)^b	0.035	2.173	0.9	17.6	(Gruner, 2003)	tip of abdomen to end of head or carapace, excl. any appendages	inferred, all insect taxa
Gastropoda	All		Dry mass	M = exp(a + b * log(L*W))	-2.75	1.59	2.1	18	(Wardhaugh, 2013)	front of labrum to tip of abdomen (excl. cerci or ovipositors) or tip of elytra (longest)	inferred, Gastropoda
Hemiptera	Acanthosomatidae		Dry mass	M = exp(a + b * log(L))	-4.784	3.075	3.2	40.23	(Sample, Cooper, Greer, & Whitmore, 1993)	From frons to tip of abdomen excluding appendages	Group specific, Hemiptera
Hemiptera	Anthocoridae		Dry mass	M = exp(a + b * log(L))	-4.784	3.075	3.2	40.23	(Sample, Cooper, Greer, & Whitmore, 1993)	From frons to tip of abdomen excluding appendages	Group specific, Hemiptera
Hemiptera	Aradidae		Dry mass	M = exp(a + b * log(L))	-4.784	3.075	3.2	40.23	(Sample, Cooper, Greer, & Whitmore, 1993)	From frons to tip of abdomen excluding appendages	Group specific, Hemiptera
Hemiptera	Ceratocombidae		Dry mass	M = exp(a + b * log(L))	-4.784	3.075	3.2	40.23	(Sample, Cooper, Greer, & Whitmore, 1993)	From frons to tip of abdomen excluding appendages	Group specific, Hemiptera
Hemiptera	Cicadellidae		Dry mass	M = exp(a + b * log(L))	-3.735	2.561	2.13	13.25	(Sample, Cooper, Greer, & Whitmore, 1993)	From frons to tip of abdomen excluding appendages	Group specific
Hemiptera	Cimicidae		Dry mass	M = exp(a + b * log(L))	-4.784	3.075	3.2	40.23	(Sample, Cooper, Greer, & Whitmore, 1993)	From frons to tip of abdomen excluding appendages	inferred, Hemiptera
Hemiptera	Cydnidae		Dry mass	M = exp(a + b * log(L))	-4.784	3.075	3.2	40.23	(Sample, Cooper, Greer, & Whitmore, 1993)	From frons to tip of abdomen excluding appendages	inferred, Hemiptera
Hemiptera	Delphacidae		Dry mass	M = exp(a + b * log(L))	-2.823	2.225	2.13	13.25	(Sample, Cooper, Greer, & Whitmore, 1993)	From frons to tip of abdomen excluding appendages	Inferred, Homoptera

Hemiptera	Dipsocoridae		Dry mass	M = exp(a + b * log(L))	-4.784	3.075	3.2	40.23	(Sample, Cooper, Greer, & Whitmore, 1993)	From frons to tip of abdomen excluding appendages	inferred, Hemiptera
Hemiptera	Enicocephalidae		Dry mass	M = exp(a + b * log(L))	-4.784	3.075	3.2	40.23	(Sample, Cooper, Greer, & Whitmore, 1993)	From frons to tip of abdomen excluding appendages	inferred, Hemiptera
Hemiptera	Eurybrachyidae		Dry mass	M = exp(a + b * log(L))	-2.823	2.225	2.13	13.25	(Sample, Cooper, Greer, & Whitmore, 1993)	From frons to tip of abdomen excluding appendages	Inferred, Homoptera
Hemiptera	Hebridae		Dry mass	M = exp(a + b * log(L))	-4.784	3.075	3.2	40.23	(Sample, Cooper, Greer, & Whitmore, 1993)	From frons to tip of abdomen excluding appendages	inferred, Hemiptera
Hemiptera	Hydrometridae		Dry mass	M = exp(a + b * log(L))	-4.784	3.075	3.2	40.23	(Sample, Cooper, Greer, & Whitmore, 1993)	From frons to tip of abdomen excluding appendages	inferred, Hemiptera
Hemiptera	Lophopidae		Dry mass	M = exp(a + b * log(L))	-2.823	2.225	2.13	13.25	(Sample, Cooper, Greer, & Whitmore, 1993)	From frons to tip of abdomen excluding appendages	Inferred, Homoptera
Hemiptera	Lygaeidae		Dry mass	M = exp(a + b * log(L))	-4.784	3.075	3.2	40.23	(Sample, Cooper, Greer, & Whitmore, 1993)	From frons to tip of abdomen excluding appendages	inferred, Hemiptera
Hemiptera	Meenoplidae		Dry mass	M = exp(a + b * log(L))	-2.823	2.225	2.13	13.25	(Sample, Cooper, Greer, & Whitmore, 1993)	From frons to tip of abdomen excluding appendages	Inferred, Homoptera
Hemiptera	Membracidae		Dry mass	M = exp(a + b * log(L))	-2.823	2.225	2.13	13.25	(Sample, Cooper, Greer, & Whitmore, 1993)	From frons to tip of abdomen excluding appendages	Inferred, Homoptera
Hemiptera	Mesoveliidae		Dry mass	M = exp(a + b * log(L))	-4.784	3.075	3.2	40.23	(Sample, Cooper, Greer, & Whitmore, 1993)	From frons to tip of abdomen excluding appendages	inferred, Hemiptera
Hemiptera	Miridae		Dry mass	M = exp(a + b * log(L))	-4.784	3.075	3.2	40.23	(Sample, Cooper, Greer, & Whitmore, 1993)	From frons to tip of abdomen excluding appendages	inferred, Hemiptera
Hemiptera	Nabidae		Dry mass	M = exp(a + b * log(L))	-4.784	3.075	3.2	40.23	(Sample, Cooper, Greer, & Whitmore, 1993)	From frons to tip of abdomen excluding appendages	inferred, Hemiptera
Hemiptera	Pentatomidae		Dry mass	M = exp(a + b * log(L))	-4.197	3.053	6.35	16.73	(Sample, Cooper, Greer, & Whitmore, 1993)	From frons to tip of abdomen excluding appendages	Group specific
Hemiptera	Reduviidae		Dry mass	M = exp(a + b * log(L))	-4.784	3.075	3.2	40.23	(Sample, Cooper, Greer, & Whitmore, 1993)	From frons to tip of abdomen excluding appendages	inferred, Hemiptera
Hemiptera	Schizopteridae		Dry mass	M = exp(a + b * log(L))	-4.784	3.075	3.2	40.23	(Sample, Cooper, Greer, & Whitmore, 1993)	From frons to tip of abdomen excluding appendages	inferred, Hemiptera
Hemiptera	Triozidea		Dry mass	M = exp(a + b * log(L))	-2.823	2.225	2.13	13.25	(Sample, Cooper, Greer, & Whitmore, 1993)	From frons to tip of abdomen excluding appendages	Inferred, Homoptera
Hymenoptera	Bethylidae		Dry mass	M = exp(a + b * log(L))	-3.336	2.104	1	12	(Gowing & Recher, 1984)	not mentioned	inferred, Hym. excl Formicidae
Hymenoptera	Diapriidae		Dry mass	M = exp(a + b * log(L))	-3.336	2.104	1	12	(Gowing & Recher, 1984)	not mentioned	inferred, Hym. excl Formicidae
Hymenoptera	Eucoilidae		Dry mass	M = exp(a + b * log(L))	-3.336	2.104	1	12	(Gowing & Recher, 1984)	not mentioned	inferred, Hym. excl Formicidae
Hymenoptera	Eupelmidae		Dry mass	M = exp(a + b * log(L))	-3.336	2.104	1	12	(Gowing & Recher, 1984)	not mentioned	inferred, Hym. excl Formicidae

Hymenoptera	Figitidae		Dry mass	M = exp(a + b * log(L))	-3.336	2.104	1	12	(Gowing & Recher, 1984)	not mentioned	inferred, Hym. excl Formicidae
Hymenoptera	Formicidae		Dry mass	M = exp(a + b * log(L))	-3.996	2.489	2	18	(Gowing & Recher, 1984)	not mentioned	Group specific
Hymenoptera	Mymariidae		Dry mass	M = exp(a + b * log(L))	-3.336	2.104	1	12	(Gowing & Recher, 1984)	not mentioned	inferred, Hym. excl Formicidae
Hymenoptera	Scelionidae		Dry mass	M = exp(a + b * log(L))	-3.336	2.104	1	12	(Gowing & Recher, 1984)	not mentioned	inferred, Hym. excl Formicidae
Hymenoptera	Specidae		Dry mass	M = exp(a + b * log(L))	-3.336	2.104	1	12	(Gowing & Recher, 1984)	not mentioned	inferred, Hym. excl Formicidae
Hymenoptera	Trichogrammatidae		Dry mass	M = exp(a + b * log(L))	-3.336	2.104	1	12	(Gowing & Recher, 1984)	not mentioned	inferred, Hym. excl Formicidae
Hymenoptera	Unidentifiable		Dry mass	M = exp(a + b * log(L))	-3.336	2.104	1	12	(Gowing & Recher, 1984)	not mentioned	inferred, Hym. excl Formicidae
Isopoda	All		Dry mass	M = exp(a + b * log(L))	-4.81	3.44	2.7	8	(Wardhaugh, 2013)	front of labrum to tip of abdomen (excl. cerci or ovipositors) or tip of elytra (longest)	Group specific, Isopoda
Isoptera	Rhinotermitidae		Dry mass	M = e^a * L^b	-5.802	3.177	3.30	5.60	(Johnson & Strong, 2000)	head to end of abdomen	inferred, Isoptera
Isoptera	Termitidae		Dry mass	M = e^a * L^b	-5.802	3.177	3.30	5.60	(Johnson & Strong, 2000)	head to end of abdomen	inferred, Isoptera
Isoptera	Unidentifiable		Dry mass	M = e^a * L^b	-5.802	3.177	3.30	5.60	(Johnson & Strong, 2000)	head to end of abdomen	inferred, Isoptera
Lepidoptera	Alucitidae		Dry mass	M = exp(a + b * log(L))	-5.909	2.959	6.26	44.62	(Sample, Cooper, Greer, & Whitmore, 1993)	frons to tip of abdomen (excl. antennae, ovipositors, wings etc.)	inferred, Lepidoptera Larvae
Lepidoptera	Arctiidae		Dry mass	M = exp(a + b * log(L))	-5.909	2.959	6.26	44.62	(Sample, Cooper, Greer, & Whitmore, 1993)	frons to tip of abdomen (excl. antennae, ovipositors, wings etc.)	inferred, Lepidoptera Larvae
Lepidoptera	Arctiidae		Dry mass	M = exp(a + b * log(L))	-3.755	2.658	5.05	20.06	(Sample, Cooper, Greer, & Whitmore, 1993)	frons to tip of abdomen (excl. antennae, ovipositors, wings etc.)	inferred, Lepidoptera
Lepidoptera	Gelechiidae		Dry mass	M = exp(a + b * log(L))	-5.909	2.959	6.26	44.62	(Sample, Cooper, Greer, & Whitmore, 1993)	frons to tip of abdomen (excl. antennae, ovipositors, wings etc.)	inferred, Lepidoptera Larvae
Lepidoptera	Geometridae		Dry mass	M = exp(a + b * log(L))	-5.493	2.625	7.66	29.50	(Sample, Cooper, Greer, & Whitmore, 1993)	frons to tip of abdomen (excl. antennae, ovipositors, wings etc.)	Group specific
Lepidoptera	Hesperiidae		Dry mass	M = exp(a + b * log(L))	-5.909	2.959	6.26	44.62	(Sample, Cooper, Greer, & Whitmore, 1993)	frons to tip of abdomen (excl. antennae, ovipositors, wings etc.)	inferred, Lepidoptera Larvae
Lepidoptera	Lasiocampidae		Dry mass	M = exp(a + b * log(L))	-5.909	2.959	6.26	44.62	(Sample, Cooper, Greer, & Whitmore, 1993)	frons to tip of abdomen (excl. antennae, ovipositors, wings etc.)	inferred, Lepidoptera Larvae
Lepidoptera	Lymantriidae		Dry mass	M = exp(a + b * log(L))	-5.909	2.959	6.26	44.62	(Sample, Cooper, Greer, & Whitmore, 1993)	frons to tip of abdomen (excl. antennae, ovipositors, wings etc.)	inferred, Lepidoptera Larvae
Lepidoptera	Noctuidae		Dry mass	M = exp(a + b * log(L))	-5.424	2.845	7.96	42.80	(Sample, Cooper, Greer, & Whitmore, 1993)	frons to tip of abdomen (excl. antennae, ovipositors, wings etc.)	Group specific

Lepidoptera	Nolidae		Dry mass	M = exp(a + b * log(L))	-5.909	2.959	6.26	44.62	(Sample, Cooper, Greer, & Whitmore, 1993)	frons to tip of abdomen (excl. antennae, ovipositors, wings etc.)	inferred, Lepidoptera Larvae
Lepidoptera	Pterophoridae		Dry mass	M = exp(a + b * log(L))	-5.909	2.959	6.26	44.62	(Sample, Cooper, Greer, & Whitmore, 1993)	frons to tip of abdomen (excl. antennae, ovipositors, wings etc.)	inferred, Lepidoptera Larvae
Lepidoptera	Pyralidae		Dry mass	M = exp(a + b * log(L))	-5.909	2.959	6.26	44.62	(Sample, Cooper, Greer, & Whitmore, 1993)	frons to tip of abdomen (excl. antennae, ovipositors, wings etc.)	inferred, Lepidoptera Larvae
Lepidoptera	Pyralidae		Dry mass	M = exp(a + b * log(L))	-5.036	3.122	2.76	40.73	(Sample, Cooper, Greer, & Whitmore, 1993)	frons to tip of abdomen (excl. antennae, ovipositors, wings etc.)	inferred, Lepidoptera
Mantodea	Mantidae		Dry mass	M = exp(a + b * log(L))	-6.340	3.010	6.00	66.00	(Wardhaugh, 2013)	front of labrum to tip of abdomen (excl. cerci or ovipositors) or tip of elytra (longest)	Group specific
Neuroptera	Chrysopidae		Dry mass	M = exp(a + b * log(L))	-4.483	2.570	3.45	54.51	(Sample, Cooper, Greer, & Whitmore, 1993)	frons to tip of abdomen (excl. antennae, ovipositors, wings etc.)	inferred, Neuroptera
Opiliones	All		Fresh mass	M = exp(a + b * log(L))	-0.899	2.984	0.57	6.9	(Höfer & Ott, 2009)	edge of prosoma (without chelicerae) to edge of opisthosoma (excl spinnerets)	inferred, Opiliones
Orthoptera	Acrididae		Dry mass	M = exp(a + b * log(L))	-3.17	2.61	2.3	33	(Wardhaugh, 2013)	front of labrum to tip of abdomen (excl. cerci or ovipositors) or tip of elytra (longest)	inferred, Orthoptera
Orthoptera	Eumastacidae		Dry mass	M = exp(a + b * log(L))	-3.17	2.61	2.3	33	(Wardhaugh, 2013)	front of labrum to tip of abdomen (excl. cerci or ovipositors) or tip of elytra (longest)	inferred, Orthoptera
Orthoptera	Gryllidae		Dry mass	M = exp(a + b * log(L))	-3.17	2.61	2.3	33	(Wardhaugh, 2013)	front of labrum to tip of abdomen (excl. cerci or ovipositors) or tip of elytra (longest)	inferred, Orthoptera
Orthoptera	Tetrigidae		Dry mass	M = exp(a + b * log(L))	-3.17	2.61	2.3	33	(Wardhaugh, 2013)	front of labrum to tip of abdomen (excl. cerci or ovipositors) or tip of elytra (longest)	inferred, Orthoptera
Plecoptera	All		Dry mass	M = a * L^b	0.0094	2.754	1.95	3.232	(Benke, Huryn, Smock, & Wallace, 1999)	Total length	Group specific
Plecoptera	Austroperlidae		Dry mass	M = a * L^b	0.0094	2.754	1.95	3.232	(Benke, Huryn, Smock, & Wallace, 1999)	Total length	Group specific
Plecoptera	Gripopterygidae		Dry mass	M = a * L^b	0.0094	2.754	1.95	3.232	(Benke, Huryn, Smock, & Wallace, 1999)	Total length	Group specific
Plecoptera	Notonemouridae		Dry mass	M = a * L^b	0.0094	2.754	1.95	3.232	(Benke, Huryn, Smock, & Wallace, 1999)	Total length	Group specific
Pseudoscorpionida	All		fresh mass	M = exp(a + b * log(L))	-1.892	2.515	0.86	2.10	(Höfer & Ott, 2009)	edge of prosoma (without chelicerae) to edge of opisthosoma (excl spinnerets)	Group specific
Psocoptera	Archipsocidae		Dry mass	M = a * (L)^b	0.014	3.115	1.50	3.15	(Gruner, 2003)	tip of abdomen to end of head or carapace, excl. any appendages	inferred, Psocoptera
Psocoptera	Caeciliidae		Dry mass	M = a * (L)^b	0.014	3.115	1.50	3.15	(Gruner, 2003)	tip of abdomen to end of head or carapace, excl. any appendages	inferred, Psocoptera
Psocoptera	Ectopsocidae		Dry mass	M = a * (L)^b	0.014	3.115	1.50	3.15	(Gruner, 2003)	tip of abdomen to end of head or carapace, excl. any appendages	inferred, Psocoptera
Psocoptera	Elipsocidae		Dry mass	M = a * (L)^b	0.014	3.115	1.50	3.15	(Gruner, 2003)	tip of abdomen to end of head or carapace, excl. any appendages	inferred, Psocoptera

Psocoptera	Epipsocidae		Dry mass	M = a * (L)^b	0.014	3.115	1.50	3.15	(Gruner, 2003)	tip of abdomen to end of head or carapace, excl. any appendages	inferred, Psocoptera
Psocoptera	Hemipsocidae		Dry mass	M = a * (L)^b	0.014	3.115	1.50	3.15	(Gruner, 2003)	tip of abdomen to end of head or carapace, excl. any appendages	inferred, Psocoptera
Psocoptera	Lepidopsocidae		Dry mass	M = a * (L)^b	0.014	3.115	1.50	3.15	(Gruner, 2003)	tip of abdomen to end of head or carapace, excl. any appendages	inferred, Psocoptera
Psocoptera	Pachytroctidae		Dry mass	M = a * (L)^b	0.014	3.115	1.50	3.15	(Gruner, 2003)	tip of abdomen to end of head or carapace, excl. any appendages	inferred, Psocoptera
Psocoptera	Psocidae		Dry mass	M = a * (L)^b	0.014	3.115	1.50	3.15	(Gruner, 2003)	tip of abdomen to end of head or carapace, excl. any appendages	inferred, Psocoptera
Psocoptera	Psyllipsocidae		Dry mass	M = a * (L)^b	0.014	3.115	1.50	3.15	(Gruner, 2003)	tip of abdomen to end of head or carapace, excl. any appendages	inferred, Psocoptera
Psocoptera	Unidentifiable		Dry mass	M = a * (L)^b	0.014	3.115	1.50	3.15	(Gruner, 2003)	tip of abdomen to end of head or carapace, excl. any appendages	inferred, Psocoptera
Schizomida	Hubbardiidae		Fresh mass	M = exp(a + b * log(L))	-2.108	3.017	0.67	36	(Höfer & Ott, 2009)	edge of prosoma (without chelicerae) to edge of opisthosoma (excl spinnerets)	inferred, hunting spiders
Symphyla	Scutegerillidae		Dry mass	M = a * (L)^b	0.035	2.173	0.9	17.6	(Gruner, 2003)	tip of abdomen to end of head or carapace, excl. any appendages	inferred, all insect taxa
Thysanoptera	Aeolothripidae		Dry mass	M = a * (L)^b	0.035	2.173	0.9	17.6	(Gruner, 2003)	tip of abdomen to end of head or carapace, excl. any appendages	inferred, all insect taxa
Thysanoptera	Phlaeothripidae		Dry mass	M = a * (L)^b	0.035	2.173	0.9	17.6	(Gruner, 2003)	tip of abdomen to end of head or carapace, excl. any appendages	inferred, all insect taxa
Thysanoptera	Thripidae		Dry mass	M = a * (L)^b	0.035	2.173	0.9	17.6	(Gruner, 2003)	tip of abdomen to end of head or carapace, excl. any appendages	inferred, all insect taxa
Thysanura	Nicoletiidae		Dry mass	M = exp(a + b * log(L))	-3.628	2.494	2.13	54.51	(Sample, Cooper, Greer, & Whitmore, 1993)	From frons to tip of abdomen excluding appendages	inferred, all insect taxa

Supplementary Table 4b. Dry-to-fresh mass conversions from the literature[12] for transformation of dry body masses (DM) (from length-dry mass regression calculations) to fresh mass (FM).

**Taxon**	**Equation FM[mg], DM[mg]**	**a**	**b**	**Reference**	**Regression specificity**
Annelida	FM = exp(a + b * log(DM))	0.9282	1.0899	(Mercer et al., 2001)	Oligochaeta
All other groups with dry-mass length-mass regressions, (see Supplementary Table S4a)	FM = exp(a + b * log(DM))	0.6111	1.0213	(Mercer et al., 2001)	Insects

Supplementary Table 5. Regression parameters for individual metabolic rate calculation from the literature[13] and unpublished data (Roswitha Ehnes). Phylogenetic model: $\ln I = \ln i_{oPG} + a_{PG} \ln M - E_{PG} (1/kT)$; Linear model: $\ln I = \ln i_o + a \ln M - E (1/kT)$. I is the metabolic rate, a is the allometric exponent, E is the activation energy, k is the Boltzmann constant, T the temperature in Kelvin (in our models taken as local mean soil temperature) and i_o a normalisation factor.

Regression group	Applied to taxa	$\ln i_o$ / $\ln i_{oPG}$	a a_{PG}	E E_{PG}	Model
Arachnida	Araneae, Opiliones, Pseudoscorpionida, Schizomida	24.581	0.565	0.709	phylogenetic
Chilopoda	Chilopoda	28.253	0.558	0.803	phylogenetic
Clitellata	Clitellata	12.442	0.801	0.443	phylogenetic
Coleoptera	Coleoptera	21.418	0.738	0.639	phylogenetic
General	Gastropoda	23.055	0.695	0.686	linear
Hymenoptera	Hymenoptera	22.013	0.742	0.668	phylogenetic
Insecta	Arachaeognatha, Blattodea, Dermaptera, Diplura, Diptera, Hemiptera, Isoptera, Lepidoptera, Mantodea, Neuroptera, Orthoptera, Plecoptera, Psocoptera, Symphyla, Thysanoptera, Thysanura	21.972	0.759	0.657	phylogenetic
Isopoda	Isopoda	23.169	0.554	0.687	phylogenetic
Progoneata	Diplopoda	22.347	0.571	0.670	phylogenetic

Supplementary Methods. Calculation of energy fluxes (*F*) from community metabolism (*X*), assimilation efficiencies (*e*), and losses to predation (*L*). *O*, *P*, *D*, *H*, *Pl* and *Dt* denote omnivores, predators, detritivores, herbivores, plants and detritus. We denote total flux to a node *I* as F_I and the flux from node *J* to *I* as F_{IJ} . For example, F_O is the total flux to omnivores and F_{OP} is the flux from predators to omnivores. Assimilation efficiencies of animal food (0.60), plant food (0.45) and detritus food (0.25)[14] are given as e_a, e_p, and e_d, respectively.

$$F_O = F_{OP} + F_{OH} + F_{OD} + F_{OPl} + F_{ODt} \quad (1)$$

We assume that predators, herbivores and detritivores each contribute to ¼ of the omnivore diet and plants and detritus equally contribute to the remaining ¼.

$$F_{OP} = F_{OH} = F_{OD} = \frac{1}{4} F_O \quad (2)$$

$$F_{OPl} = F_{ODt} = \frac{1}{8} F_O \quad (3)$$

The community metabolism *X* of a node is given as

$$X = (F \cdot e) - L \, . \quad (4)$$

Thus, the energy entering the omnivore node is given as

$$X_O + L = e_a \cdot (F_{OP} + F_{OH} + F_{OD}) + e_p \cdot F_{OPl} + e_d \cdot F_{ODt} = \left(\frac{3}{4} e_a + \frac{1}{8} e_p + \frac{1}{8} e_d\right) \cdot F_O \, , \quad (5)$$

where equations 2 and 3 were used to replace single fluxes with the fraction of the overall flux.

The efficiency with which omnivores assimilate resources is

$$e_O = \left(\frac{3}{4} e_a + \frac{1}{8} e_p + \frac{1}{8} e_d\right) . \quad (6)$$

Now, to express F_O, e_O needs to be replaced by equation 6, which yields

$$F_O = \frac{1}{e_O} \cdot \left(X_O + \frac{F_P}{3}\right) . \quad (7)$$

The equation for predators is similar but with the e_a assimilation efficiency, yielding

$$F_P = \frac{1}{e_a} \cdot \left(X_P + \frac{F_O}{4}\right) . \quad (8)$$

We then solve for F_P by inserting equation 7 into 8:

$$F_P = \frac{12 \cdot e_O \cdot X_P + 3 \cdot X_O}{12 \cdot e_a \cdot e_O - 1} . \quad (9)$$

Now we calculate F_O using equation 7, and, with F_P and F_O we can calculate F_H and F_D using equations

$$F_H = \frac{1}{e_p} \cdot \left(X_H + \frac{F_P}{3} + \frac{F_O}{4}\right) \tag{10}$$

and

$$F_D = \frac{1}{e_d} \cdot \left(X_D + \frac{F_P}{3} + \frac{F_O}{4}\right). \tag{11}$$

Supplementary References:

1. Peters, R. H. *The Ecological Implications of Body Size. Cambridge Stud. Ecol.* **Cambridge,** 329 (1983).

2. Johnson, M. & Strong, A. Length weight relationships of Jamaican arthropods. *Entomol. News* **111,** 270–281 (2000).

3. Benke, A. C., Huryn, A. D., Smock, L. A. & Wallace, J. B. Length-mass relationships for freshwater macroinvertebrates in North America with particular reference to the southeastern United States. *J. North Am. Benthol. Soc.* **18,** 308 (1999).

4. Hale, C. M., Reich, P. B. & Frelich, L. E. Allometric equations for estimation of ash-free dry mass from length measurements for selected European earthworm species (Lumbricidae) in the Western Great Lakes region. *Am. Midl. Nat.* **151,** 179–185 (2004).

5. Höfer, H. & Ott, R. Estimating biomass of Neotropical spiders and other arachnids (Araneae, Opiliones, Pseudoscorpiones, Ricinulei) by mass-length regressions. *J. Arachnol.* **37,** 160–169 (2009).

6. Sample, B. E., Cooper, R. J., Greer, R. D. & Whitmore, R. C. Estimation of insect biomass by length and width. *Am. Midl. Nat.* **129,** 234 (1993).

7. Wardhaugh, C. W. Estimation of biomass from body length and width for tropical rainforest canopy invertebrates. *Aust. J. Entomol.* **52**, *291-298* (2013).

8. Gowing, G. & Recher, H. F. Length-weight relationships for invertebrates from forests in south-eastern New South Wales. *Austral Ecol.* **9,** 5–8 (1984).

9. Edwards, R. Estimating live spider weight using preserved specimens. *J. Arachnol.* **24,** 161–166 (1996).

10. Lang, A., Krooss, S. & Stumpf, H. Mass-length relationships of epigeal arthropod predators in arable land (Araneae, Chilopoda, Coleoptera). *Pedobiologia.* **41,** 327–333 (1997).

11. Gruner, D. S. Regressions of length and width to predict arthropod biomass in the Hawaiian Islands. *Pacific Sci.* **57,** 325–336 (2003).

12. Mercer, R. D., Gabriel, A. G. A., Barendse, J., Marshall, D. J. & Chown, S. L. Invertebrate body sizes from Marion Island. Antarct. Sci. 13, 135–143 (2001).

13. Ehnes, R., Rall, B. & Brose, U. Phylogenetic grouping, curvature and metabolic scaling in terrestrial invertebrates. *Ecol. Lett.* **14,** 993–1000 (2011).

14. de Ruiter, P. C., Veen, J. a., Moore, J. C., Brussaard, L. & Hunt, H. W. Calculation of nitrogen mineralization in soil food webs. *Plant Soil* **157,** 263–273 (1993).
